# Scientific risk communication about controversial issues influences public perceptions of scientists' political orientations and credibility

**DOI:** 10.1098/rsos.170505

**Published:** 2018-02-21

**Authors:** Emily Vraga, Teresa Myers, John Kotcher, Lindsey Beall, Ed Maibach

**Affiliations:** Center for Climate Change Communication, Department of Communication, George Mason University, Fairfax, VA, USA

**Keywords:** science communication, risk communication, motivated reasoning, political ideology, perceptions of scientists

## Abstract

Many scientists communicate with the public about risks associated with scientific issues, but such communication may have unintended consequences for how the public views the political orientations and the credibility of the communicating scientist. We explore this possibility using an experiment with a nationally representative sample of Americans in the fall of 2015. We find that risk communication on controversial scientific issues sometimes influences perceptions of the political orientations and credibility of the communicating scientist when the scientist addresses the risks of issues associated with conservative or liberal groups. This relationship is moderated by participant political ideology, with liberals adjusting their perceptions of the scientists' political beliefs more substantially when the scientist addressed the risks of marijuana use when compared with other issues. Conservatives' political perceptions were less impacted by the issue context of the scientific risk communication but indirectly influenced credibility perceptions. Our results support a contextual model of audience interpretation of scientific risk communication. Scientists should be cognizant that audience members may make inferences about the communicating scientist's political orientations and credibility when they engage in risk communication efforts about controversial issues.

## 1. Introduction

Most Americans have little familiarity with scientists, or with science. Although Americans largely have favourable perceptions of scientists and their contributions to societal quality of life, their knowledge about scientists and the scientific process remains low [1,2]. Instead, most Americans learn about scientists only through interaction with the media—specifically, through popular culture and news coverage of scientific issues and research [2,3]. As such, their perceptions of scientific motives, goals and political orientations are likely to be relatively superficial, resulting from heuristic processing of scientific information [4,5].

At the same time, political polarization in the United States is on the rise for a range of scientific issues, with division occurring along party lines on issues such as climate change, nuclear power, marijuana use and fracking [6–10]. Perceptions of scientists—including trust in the scientific community and perceptions of their ideological leaning—have also become more divided, with conservatives generally reporting more scepticism towards scientists, especially those scientists focused on certain environmental and health impacts [2,11,12].

These trends can present a conundrum for scientists interested in engaging in risk communication with the public. On the one hand, informing the public about social risks is an important component of science communication efforts [13,14]. On the other, such risk communication may shape perceptions of the scientist's political beliefs, particularly when the scientist publicly addresses a contentious or politicized scientific issue. It is the latter possibility that we examine in this paper by testing whether the issue context for risk communication—even without explicit endorsement of specific solutions to reduce the risk—shapes perceptions of the political orientations of scientists.

Moreover, perceptions of a scientist's political orientations may also have consequences for ratings of their credibility. Although past research has not directly examined the mediating role of perceptions of scientists' political ideology or party affiliation, perceived value similarity [15,16] and perceptions of communicator bias [17,18] are both important predictors of communicator trust and credibility. If individuals come to see a scientist as political in their orientation, it may shape their perceptions of the scientist's credibility.

The potential for politicized attributions of scientists engaging in risk communication is especially likely to occur when the communicating scientist is providing information that is incongruent with an individual audience member's personal beliefs or worldview. In these cases, a ‘contextual effects’ or motivated reasoning model of processing suggests that people will attempt to discredit the scientist by ascribing a political motive to their efforts, which is problematic if the association between scientists and a particular political or ideological group undermines confidence in science and reduces the likelihood that scientific evidence will be considered in democratic decision-making [9,19–21]. Therefore, scientists engaging in risk communication on a contentious scientific issue that generally disagrees with one's political beliefs (i.e. discussing an issue where risk perceptions are polarized across political groups and the issue is seen as favouring an opposing political group) are likely to create larger shifts in perceptions of the political orientations of the scientist than scientists communicating about non-controversial scientific issues.

Our study tests these propositions using an experiment conducted with a national sample of American adults in 2015. Participants viewed a scientist engaging in risk communication on one of four scientific issues—two of low controversy (severe weather and flu) and two of high controversy (climate change and marijuana use)—in which no information is provided about solutions. We expect that even when the scientist is engaging in the most basic form of risk communication—simply highlighting the potential dangers of an issue without advocating for any particular solution—the issue being addressed will shape perceptions of the scientist's political orientations. Specifically, when a scientist highlights the dangers of an issue seen as risky primarily by conservatives (marijuana use), it will lead to estimates of the scientist as more conservative—and when a scientist addresses an issue seen as risky primarily by liberals (climate change), it will create perceptions of the scientist as liberal. In addition, we expect that these perceptions of a scientist's political beliefs will mediate the relationship between issue context and perceptions of the scientist's credibility. We anticipate that this process will be exacerbated by participants' political ideology, with attributions of scientific political orientations being strongest when a scientist is communicating risk on an issue seen as affiliated with the opposing political group. As such, this study explores one potential consequence of basic risk communication about contentious scientific issues, even without advocacy efforts.

### 1.1. Public perceptions of scientists

Americans generally have favourable perceptions of science, saying that science has improved the quality of life in the realms of medicine, food and the environment [1,2], and that a career as a scientist is prestigious in comparison to other fields [22]. But in spite of this generalized positive view of science, the US public does not know much about the specifics of how science occurs, which includes a lack of awareness that scientific research is conducted in their region of the country, an inability to recognize large federal scientific research agencies like the National Institutes of Health and the National Science Foundation, and an inability to name any living scientist [22–24]. Scientists have decried the public's levels of scientific knowledge, arguing that this lack of knowledge leads to expectations for quick solutions to difficult problems and prevents the ability to distinguish well-founded from not-well-founded scientific claims [1,2].

This limited knowledge among the American public may be linked to a lack of direct experience with science. Americans encounter information about science and scientists primarily through news reporting [2] or through depictions of scientists in movies and television shows. Entertainment media portrayals of scientists are infrequent, and typically stereotyped—showing scientists as white males who sometimes have mixed motives [3,25,26]. Americans' top-of-the-mind perceptions of scientists are largely consistent with these pop-culture depictions; scientists are thought of as white, male, nerds [27]. Some research has directly linked media exposure to perceptions of scientists [9,25,28], although these effects may be nuanced [3,29].

This research shows that Americans' perceptions of scientists are based more on cultural understanding of what a scientist ‘is’ than on personal (or even mediated) knowledge about actual scientists and how they conduct their work. Research suggests that for issues about which people have low amounts of information or that aren't salient to an individual's life, judgements tend to be made based on easily available information; an information-processing strategy labelled heuristic processing [30,31]. It seems likely, then, that Americans' views of scientists and science are based on such heuristic processing, indicating large potential for relatively superficial cues to influence perceptions. In this study, we examine how one such cue—the issue context which a scientist is addressing in their risk communication with the public—influences perceptions about the political leaning (or neutrality) of the scientist engaging in such risk communication.

Additionally, we have reason to believe that these perceptions of the political leaning of the scientist will also shape perceptions of the scientist's credibility. Research has established that people assess how similar a communicator is to themselves in terms of identity, for example, [15,16], and tend to find similar individuals more trustworthy. Likewise, being unbiased has long been an indicator for credibility across a range of disciplines, including news media [32,33] and specifically for perceptions of scientists [34]—and in one case which specifically examined the bias of ‘pursuit of a liberal agenda’ [35], perceived credibility was explicitly linked to perceptions of political ideology. Likewise, some qualitative evidence also suggests that perceived ideology of scientists may be a factor that influences credibility. Interview participants reported that they are sceptical of the scientific community because of the belief that some scientists actively promote a particular agenda [36]. Therefore, we expect a relationship to emerge between perceptions of a scientist's political beliefs and their credibility.

### 1.2. The role of the public's political beliefs on perceptions of scientists

Yet while perceptions of a scientist's political leanings may be broadly influential, these perceptions are also likely to intersect with an individual's own political beliefs. Through motivated reasoning [37,38], audience members' political beliefs can influence their perceptions of scientists' political orientation through one of two ways: a ‘main effects model’ or a ‘contextual effects model’.

The first mechanism by which political beliefs can influence perceptions of scientists' political orientations is the ‘main effects model’. In this case, audience members' political ideology influences their perceptions of scientists' political leanings as a whole, independent of the issue about which the scientist is communicating. Indeed, there is evidence that, when compared with liberals, conservatives are predisposed to distrust and reject a wide variety of scientific evidence [11,23], possibly because of innate differences in psychological traits [39]. This lower level of trust implies that conservatives are more likely to view scientists as liberal because individuals tend to perceive the values of those they distrust as dissimilar to their own values [15]. While few studies have directly examined the perceived ideology of scientists, one recent survey found that while a majority of Americans view scientists as neither liberal nor conservative, individuals on the ends of the political spectrum (i.e. conservative Republicans and liberal Democrats) were more likely to view scientists as a liberal group compared to those with more moderate political views [40]. Among these strong partisans, the tendency to view the world in politicized terms has translated into perceptions of scientists as political. Moreover, sustained conservative efforts to paint scientists as ‘liberal’ have been moderately successful among partisans, much like efforts to decry liberal bias in the press [11,41,42]. This perception of liberal bias among scientists is more pronounced among conservative Republicans than liberal Democrats [40]. This suggests that conservatives will be more prone to assess scientists as liberal than liberals, regardless of the communication efforts of the scientists.

We term the second competing mechanism the ‘contextual effects model’. Under this model, an individual's political orientation influences perceptions of scientists' ideology differently within each specific issue domain. This perspective predicts that individuals will be motivated to distrust risk information from scientists when it has policy implications incongruent with their ideological beliefs [9,20,43–45]. This view is consistent with studies that have found political ideology to be a strong predictor of beliefs about certain scientific issues (e.g. climate and energy), and a poor predictor on others (e.g. food safety and space travel [2,45]). It also accords with research showing that political ideology influences generalized trust in scientists differently from trust towards scientists regarding specific issues, such as climate change [23].

Experimental evidence in support of the contextual effects model has found that people with an egalitarian–communitarian worldview (predominant among political liberals) tend to be more distrustful of scientists who say that climate change and nuclear waste disposal both present a low risk and therefore warrant limited societal action, a pattern that is reversed among people with a hierarchical–individualist worldview, predominant among political conservatives [20]. Similarly, Nisbet and colleagues [9] found that liberals and conservatives exhibited similar patterns of distrust towards the scientific community when they were presented with factual information that was incongruent with their ideological beliefs—for liberals, information on the issues of nuclear power and fracking, and for conservatives, when addressing climate change or evolution.

Although there is some evidence for both the main effects and contextual effects models, our primary interest in this study is in testing the contextual model of ideological effects on perceptions of scientists among political partisans. The motivated reasoning literature suggests people respond strongly to protect their beliefs when exposed to information that disagrees with their worldview [9,37,43]. This process should lead political ideology to condition an individual's response to scientific communication on controversial issues—both in shaping perceptions of the scientist's ideology and in translating those perceptions of that ideology into credibility perceptions—especially when seeing a scientist adopting an incongruent position.

### 1.3. The politicization of science and scientists

The potential for a scientist's risk communication to influence audience members' perceptions of the scientist's political orientation is rooted in the increasingly politicized context of American public dialogue in general, and science communication specifically. Perceptions of many scientific issues are now divided among political and ideological lines [8–10], which may lead the public to assume—in the absence of additional information—that a scientist communicating about that issue is necessarily associated with one political orientation, or the other. For example, the issue of climate change and its risks to human populations is generally seen as a liberal issue, garnering greater support among liberals and Democrats when compared with conservatives and Republicans [8,46]. By contrast, conservatives tend to believe that marijuana use is particularly risky and problematic for society, and are thus less supportive of its legalization than liberals or moderates [10]. Therefore, a scientist who is publicly addressing the risks of these issues may also be seen as more likely to align with the political orientations of those concerned about that issue. And given motivated reasoning, members of the aligned party will see the scientist as more credible as a result of their updated assessments of the scientist's political beliefs—and vice versa for an unaligned scientist addressing an incongruent issue.

We test these questions on perceptions of the political ideology and the party affiliation of the scientist. While the link between political ideology and party affiliation has grown in recent years among members of the public [6,47,48], political ideology remains an inherently less visible label for attributing bias, especially in a media environment in which party affiliation is often explicitly assigned to a source. Therefore, it is important to test whether perceptions of a scientist's political ideology or party affiliation may differ as a result of the issue context.

### 1.4. Hypotheses and research questions

*H1*: A scientist will be seen as (a) more conservative and Republican in their political orientations when discussing the risks of marijuana use and (b) more liberal and Democratic when discussing the risks of climate change, when compared with non-controversial issues (e.g. the flu and severe weather).
*H2*: Issue context will have an indirect effect on perceptions of the communicating scientist's credibility via perceptions of the communicating scientist's political orientations.
*H3*: The effects of risk communication on perceptions of the communicating scientist's political orientations will depend on participants' political ideology, such that: (a) when a scientist discusses the risks of marijuana use, both liberals and conservatives will view the scientist as more conservative than when a scientist discusses the risks of non-controversial issues; however, this effect will be stronger for liberals than conservatives; and (b) when a scientist discusses the risks of climate change, both liberals and conservatives will view the scientist as more liberal than when a scientist discusses the risks of non-controversial issues; however, this effect will be stronger for conservatives than liberals.
*H4*: The indirect effects of the issue context on the communicating scientist's credibility via perceptions of their political orientations will depend on participants' political ideology, such that (a) when a scientist discusses the risks of marijuana use, it will have a negative indirect effect on his credibility among liberals and a positive indirect effect among conservatives via perceptions of his political orientations; and (b) when a scientist discusses the risks of climate change, it will have a negative indirect effect on his credibility among conservatives and a positive indirect effect among liberals via perceptions of his political orientations.

## 2. Methods

### 2.1. Experimental design

We used an experimental design to test our hypotheses. All participants read an excerpt from an op-ed in *USA Today* by Dr Dave Wilson (a fictional scientist created for this study),^1^ wherein he explicitly addressed the risks revealed by recent scientific studies on one of four issues: marijuana use, climate change, flu or severe weather.^2^ In this manuscript, we compare the issue context for a conservative issue (marijuana use), a liberal issue (climate change), and two non-polarized issues (severe weather and flu).^3^ Scientific communication about the risks of marijuana use was selected as a ‘conservative’ issue, given that Republicans are much less supportive of legalization (39%) than Democrats (63%) or Independents (58%), and more likely to see marijuana as harmful to society in comparison to alcohol [10]. We selected scientific communication about the risks of climate change as a ‘liberal’ issue: Democrats are more worried about the risks of climate change (75%) than Republicans (36%) or Independents (53%), and more supportive of government action like regulating carbon dioxide to address it [46]. The risks of the flu and severe weather were selected as less polarizing issues within similar scientific domains (e.g. health, earth science), and both serve as relevant comparison categories for our experimental hypotheses. For the marijuana and flu conditions, Dr Wilson was introduced as a recognized international expert in public health, and for severe weather and climate change he was identified as an expert in meteorology (exact wording of the experimental stimuli and the dataset with the variables analysed in this study can be found in the electronic supplementary material). This study received approval from the Institutional Review Board at George Mason University and all participants gave their informed consent before taking part in the study.

A pretest confirmed our operationalization of these issues. For the pretest, we used a national sample of adults from Qualtrics^4^ of *N* = 205 in July of 2015. Participants viewed all four of the same excerpts from the experiment regarding the risks of the flu, marijuana use, severe weather and climate change to the American public in random order. After viewing the excerpt, they rated how controversial the issue was among Americans on a five-point scale. The results suggest that climate change (*M* = 3.48, s.d. = 1.00) and marijuana use (*M* = 3.48, s.d. = 1.08) were both seen as more controversial than the flu (*M* = 2.20, s.d. = 1.05, *p*-values < 0.001 from paired sample t-tests) or severe weather (*M* = 2.33, s.d. = 1.18, *p*-values < 0.001 from paired sample *t*-tests).

### 2.2. Sample

In October and November of 2015, we surveyed a quota sample of adult Americans who were members of an online panel maintained by Qualtrics and were selected to approximate national US representation on gender, age and education. A total of 808 participants received the stimuli relevant to this paper (approximately 200 participants per issue context condition).^5^ Participants were in the age range of 18–87, with an average age of 46 years. Of the participants, 49% were male (51% female); 11% of participants had less than a high school education, 30% had a high school education, 31% had some college, 18% were college graduates and 19% had a post-graduate education. Age, education and gender did not differ between the ‘issue context’ experimental conditions.

### 2.3. Measures

#### 2.3.1. Perceptions of Dr Wilson's political orientations

Two measures were used to capture perceptions of the political orientations of the communicating scientist. First, participants rated Dr Wilson's political ideology (*M* = 3.14, s.d. = 1.00) on a five-point scale, ranging from very conservative to very liberal. Next, participants rated the party affiliation of Dr Wilson (*M* = 3.95, s.d. = 1.53) on a seven-point scale, ranging from Strong Republican to Strong Democrat.

#### 2.3.2. Perceptions of Dr Wilson's credibility

We adapted a series of nine items to measure credibility [49]. Participants rated Dr Wilson's expertise, intelligence, competence, trustworthiness, sensitivity, sincerity, concern for society, care for society and honesty on a series of eight-point semantic differentials, with ‘not at all [characteristic]’ to ‘extremely [characteristic].’ These items were averaged to create an index (*α* = 0.90, *M = *5.67*,* s.d.*= *1.36). Unfortunately, after conducting the experiment, we noticed that a programming error meant that although there were eight radio buttons that participants could select for each characteristic, the scale was labelled from 1 to 7 (with the labels appearing between the radio buttons). We include a screenshot in the electronic supplementary material, appendix to demonstrate this problem. While we acknowledge this limitation, we believe that semantic differentials measure participants' overall attitudes based on the proximity of their selection of the anchors (e.g. extremely intelligent versus not at all intelligent), rather than a numerical value. Moreover, this error is consistent across experimental conditions, suggesting differences are due to the treatment. Despite the programming error, we believe this measure still reflects the degree to which participants find Dr Wilson credible.

#### 2.3.3. Participants' political ideology

At the end of the survey, participants were asked to rate their own political ideology on the same five-point scale, ranging from very liberal to very conservative (*M* = 3.05, s.d. = 1.10). This scale was split into three groups: with those who said they were somewhat or very liberal classified as ‘liberals’ (*N* = 219, 27.1%), those who said they were somewhat or very conservative classified as ‘conservatives’ (*N* = 248, 30.7%) and those who said they were moderate, middle of the road classified as moderates (*N* = 341, 42.2%).^6^

## 3. Results

### 3.1. Issue context

To test our first hypotheses, we conducted ordinary least-squares regression. We used dummy coding for the experimental conditions in order to generate comparisons between all of the issue contexts; results are presented in table 1.

**Table 1.** Coefficients predicting Dr Wilson and scientists' political orientation from the issue context. Entries are unstandardized regression coefficients. Each model was run twice to generate all comparisons: once with the reference category of severe weather and again with the reference category of flu. The intercept is from the model with severe weather as the reference category. In these models, a higher number indicates a more liberal or Democratic orientation.

	Dr Wilson's ideology	Dr Wilson's party
intercept	0.181**	0.488**
marijuana		
versus flu	−0.463***	−0.752***
versus severe weather	−0.471*	−1.065***
climate change		
versus flu	0.249*	0.567***
versus severe weather	0.241*	0.254^+^
liberal (versus conservative)	0.016	−0.458**
moderate (versus conservative)	0.056	−0.277*

***p < 0.001.

**p < 0.01.

*p < 0.05.

^+^*p* < 0.10.

**Figure 1.**
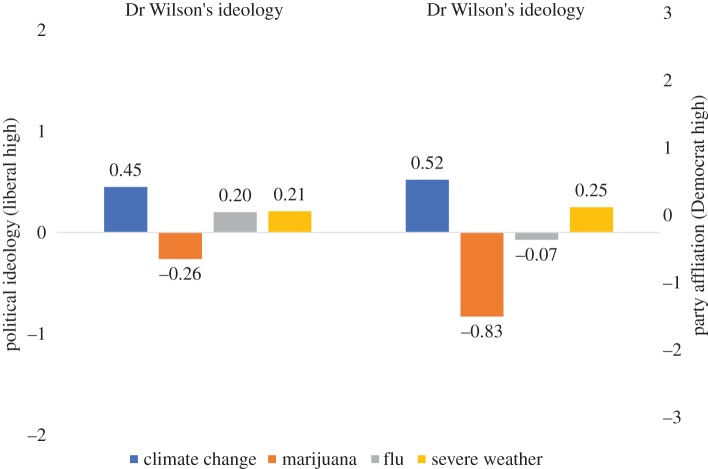
Main effect of issue context on perceptions of Dr Wilson's political orientation. This figure shows estimated marginal means by condition. See table 1 for coefficients representing the significance of differences between conditions.

We begin by testing H1a, which examined how the issue Dr Wilson addressed in his public risk communication influenced perceptions of his political orientations. The data supported H1a: Dr Wilson was perceived as more *conservative* (*p*-values < 0.001) and *Republican* (*p*-values < 0.001) when he addressed the risks of marijuana use compared to the non-controversial issue contexts (e.g. the flu or severe weather events) (figure 1).

Next, we tested H1b, which predicted that Dr Wilson would be seen as more liberal and Democratic when his editorial focused on the risks of climate change versus non-controversial issues. The data largely supported H1b: Dr Wilson was seen as significantly more *liberal* (*p* < 0.01) and *Democratic* (*p* < 0.01) when the issue context of his editorial was climate change compared to the flu issue context. However, the difference between the climate change and severe weather was less clear: Dr Wilson was seen as more *liberal* (*p* < 0.05) and marginally more *Democratic* (*p* < 0.10) when addressing climate change compared to severe weather.

We test H2, which explored whether the issue context produced an indirect effect on perceptions of Dr Wilson's credibility by shifting perceptions of his political orientations. We used the PROCESS macro for SPSS (Model 4) to test this mediated relationship [50]. We ran these analyses twice, once using flu as the reference group and secondly with severe weather as the reference group to generate all comparisons (table 2).

**Table 2.** Testing mediation of the issue context on credibility, through political orientation of Dr Wilson for all participants. Entries are the point estimate for the indicated relationship. In these models, a higher number for perceptions of Dr Wilson's political orientation indicates a more liberal or Democratic orientation.

		credibility	
	Dr Wilson's political orientation	direct effect	indirect effect through political orientation
political orientation: Dr Wilson's ideology			
marijuana versus flu	−0.46***	−0.56***	0.03
marijuana versus severe weather	−0.46***	−0.31***	0.03
climate change versus flu	0.25*	−0.33*	−0.02
climate change versus severe weather	0.24*	−0.08	−0.02
Dr Wilson's ideology		−0.07	
political orientation: Dr Wilson's party			
marijuana versus flu	−0.76***	−0.56***	0.03
marijuana versus severe weather	−1.08***	−0.31*	0.03
climate change versus flu	0.59***	−0.33*	−0.02
climate change versus severe weather	0.27^+^	−0.08	−0.01
Dr Wilson's party		−0.03	

***p < 0.001.

**p < 0.01.

*p < 0.05.

^+^*p* < 0.10.

We find limited support for these hypotheses. In no case do perceptions of Dr Wilson's political ideology or party affiliation mediate the effect of issue context on evaluations of Dr Wilson's credibility. Instead, we consistently find that when Dr Wilson communicates about the risks of a controversial issue—either marijuana use or climate change—his credibility declines compared to when he discusses a non-controversial issue—either the flu or severe weather, independently of the effect of issue context on Dr Wilson's political orientation. The only exception is the comparison between climate change and severe weather, which is insignificant. Therefore, we find no support for H2a and H2b about the mediated effects of Dr Wilson's perceived political orientations on his credibility.

Overall, these results suggest that the issue context does matter for politicized perceptions of scientists who engage in risk communication, as well as for his or her credibility. When Dr Wilson discussed a politicized scientific issue like marijuana or climate change, he was seen as having political orientations that matched public divides on these issues. In other words, Dr Wilson was viewed as more Republican and conservative when addressing the risks of marijuana use and more Democratic and liberal when discussing climate change. The effects on political orientations appear stronger in magnitude when the scientist is addressing the risks of marijuana use (a conservative issue) versus the risks of climate change (a liberal issue)—in comparison to the less politicized issue contexts of severe weather and the flu. Likewise, issue context directly reduces Dr Wilson's credibility when addressing controversial issues, but this relationship is not mediated by perceptions of his political orientations.

### 3.2. The moderating effects of participant ideology

However, the effects of issue context on perceptions of scientists' political orientations may be conditioned by the political ideology of the participants in this study, as predicted by H3a and H3b. To test these hypotheses, we compared the size of the difference between different issue conditions for liberal versus conservative individuals in our study, using PROCESS macros for SPSS [50] to generate multi-categorical interactions between issue context and liberal versus conservative political ideology (moderates were excluded from this analysis as we did not have specific predictions for this group; those interested can see the coefficients among moderates in electronic supplementary material, appendix S4). The results are presented in table 3.

**Table 3.** Summary of results for magnitude of differences in the effect of condition, between liberals and conservatives. Entries in the liberal and conservative columns are the unstandardized regression coefficients generated from the PROCESS macro [50]; they represent the simple effect of the issue context comparison for the ideology noted. Each model was run twice to generate comparisons: once with the reference category of flu and again with severe weather. The ‘diff.’ column is the significance of the difference in the coefficients, as measured by the interaction term between the comparison and liberal versus conservative ideology; significant differences in the ‘diff.’ column indicate that the difference in magnitude between the liberal and conservative coefficients for the comparison is not likely to be due to chance.

	Dr Wilson's ideology^a^			Dr Wilson's party^b^		
	liberal	conservative	diff.	liberal	conservative	diff.
climate change						
versus flu	0.008	0.454*	n.s.	0.554^+^	0.433	n.s.
versus severe weather	−0.051	0.454*	n.s.	−0.077	0.508^+^	n.s.
marijuana						
versus flu	−1.048***	−0.432*	*	−1.238***	−0.934**	n.s.
versus severe weather	−1.107***	−0.432*	*	−1.869***	−0.859**	*

^a^Liberal was coded high for political ideology. Therefore, positive coefficients indicate a more liberal score and negative coefficients indicate a more conservative score.

^b^Democrat was coded high for political party. Therefore, positive coefficients indicate a more Democratic score and negative coefficients indicate a more Republican score.

***p < 0.001.

**p < 0.01.

*p < 0.05.

^+^*p* < 0.10.

**Figure 2.**
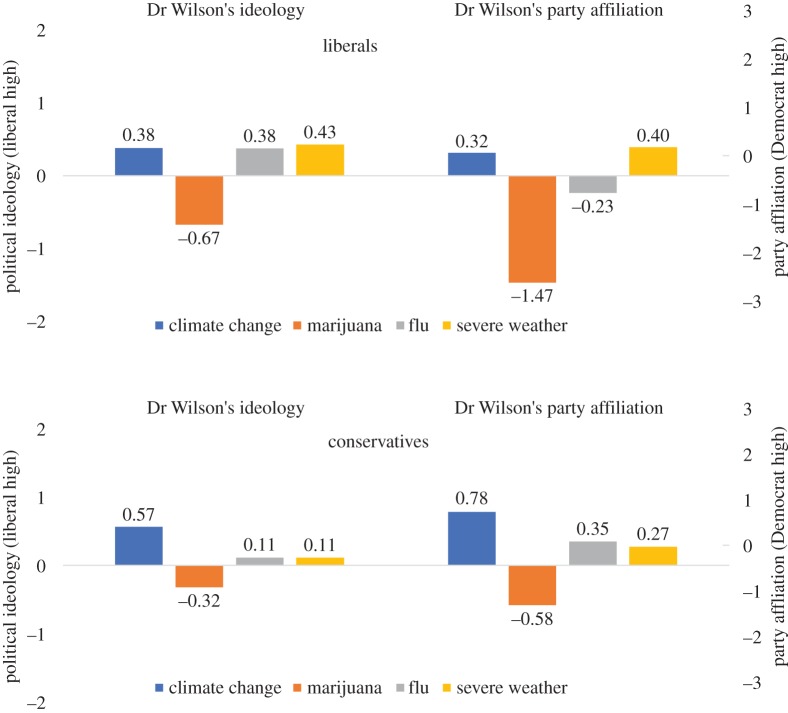
Effects of issue context on perceptions of Dr Wilson's political orientations, comparing liberals and conservatives. This figure shows estimated marginal means, by condition. See table 3 for coefficients representing the significance of differences between conditions.

H3a proposed that, compared to liberals, conservatives would be more likely to rate Dr Wilson as more *liberal* and *Democratic* when Dr Wilson addressed the risks of climate change in his editorial compared to either the flu or severe weather. We found no support for these hypotheses (see table 3; see also figure 2), as no significant differences in the size of effects between liberals and conservatives emerged when Dr Wilson discussed climate change, in comparison to the non-polarized issues of flu and severe weather.

Moving to H3b, which predicted that, compared to conservatives, liberals would experience stronger effects on their perceptions of Dr Wilson's political orientations in the marijuana issue context, we found more support for our hypotheses (see table 3; see also figure 2). Specifically, while liberals (*p*-values < 0.001) and conservatives (*p*-values < 0.05) both viewed Dr Wilson as more *conservative* in the marijuana condition relative to the flu and severe weather conditions, this effect was significantly larger among liberals compared to conservatives (*p*-values < 0.05). Similarly, compared to the severe weather condition, the marijuana issue context produced perceptions of Dr Wilson as more *Republican* among both liberals (*p* < 0.001) and conservatives (*p* < 0.01)—but this effect was significantly stronger among liberals than conservatives (*p* < 0.05); a difference that is not significant comparing the marijuana to flu conditions. These results largely support H3b—shifts in perceptions of Dr Wilson's political orientations as conservative or Republican in the marijuana issue context compared to other issue contexts were stronger for liberal than for conservative participants.

Finally, we tested H4a and H4b, which hypothesized a moderated mediation model, with participants' political ideology moderating the relationship among issue context, perceptions of Dr Wilson's political orientations and perceptions of his credibility. To test these hypotheses, we used the SPSS PROCESS macro (Model 59), which enters participant political ideology as a moderator of each of the three relationships [50]. We ran these models multiple times to generate the appropriate comparisons, which we report in table 4.

**Table 4.** Testing the moderated mediation of the issue context on credibility, through political orientation of Dr Wilson, as moderated by participant's political ideology. Entries are the point estimate for the indicated relationship.

		credibility		
	Dr Wilson's political orientation		conditional indirect effects	
	issue × participant ideology	Dr Wilson's political orientation × participant ideology	conservative	liberal
political orientation: Dr Wilson's ideology				
marijuana versus flu	−0.62*	0.90***	0.22*	−0.40***
marijuana versus severe weather	−0.67*		0.22*	−0.42**
climate change versus flu	−0.45		−0.23*	0.00
climate change versus severe weather	−0.51^+^		−0.23*	−0.02
political orientation: Dr Wilson's party				
marijuana versus flu	−0.30	0.40***	0.25***	−0.16*
marijuana versus severe weather	−1.01*		0.23*	−0.25*
climate change versus flu	0.12		−0.12	0.07
climate change versus severe weather	−0.59		−0.14^+^	−0.01

***p < 0.001.

**p < 0.01.

*p < 0.05.

^+^*p* < 0.10.

Across these analyses, we find consistent support for H4a, which predicted that the issue context of marijuana use will produce higher credibility ratings among conservatives and lower credibility ratings among liberals via the shift in perceptions of Dr Wilson's political orientations compared to either non-controversial issue. Specifically, when Dr Wilson discussed the risks of marijuana use, his credibility increased among conservative participants, through perceptions of his political orientation (both political ideology and party affiliation), in comparison to discussing the risks of flu or severe weather. Likewise, when Dr Wilson discussed the risks of marijuana use, his credibility decreased among liberals, through perceptions of his political orientation, in comparison to discussing the risks of flu or severe weather, as predicted.

However, there is only mixed support for H4b, which predicted that the issue context of climate change will produce lower credibility ratings among conservatives and higher credibility ratings among liberals via the shift in perceptions of Dr Wilson's political orientations compared to either non-controversial issue. Among conservatives, there is a mediated impact of shifts in the hypothesized direction in perceptions of Dr Wilson's political orientations on his credibility in three out of four tests (climate change versus flu and severe weather when predicting Dr Wilson's ideology, and marginally versus severe weather when predicting Dr Wilson's party). This relationship, however, does not appear to be mediated by Dr Wilson's party affiliation when comparing climate change versus the flu, although the effect is directionally consistent. Furthermore, there are no mediated effects of Dr Wilson's political orientations on his credibility among liberals when comparing climate change to either the flu or severe weather.

These analyses suggest that participants' political ideology complicates their response to a scientist communicating about risk, depending on the issue being discussed. Broadly speaking, these analyses support a contextual effects model—the significant positive interaction between perceptions of Dr Wilson's ideology and participants' ideology on credibility ratings suggests the relationship to credibility is stronger when the scientist's and participant's ideology are aligned. Likewise, the indirect effect of the marijuana issue context appears stronger than the climate change issue context, as liberals showed no indirect effects of the climate change issue context on perceptions of Dr Wilson's credibility when compared with either non-controversial issue.^7^

## 4. Discussion

The public depends on scientists to accurately convey information about the risks associated with a range of scientific issues. Such risk communication may come with dangers of creating or reinforcing perceptions of scientists as affiliated with specific political beliefs, especially given low levels of familiarity with scientists and rising politicization for many scientific issues [5,51–54]. Such an assumption is especially troublesome if perceptions of political beliefs also then shape evaluations of the scientist's credibility. In this study, we argue that the issue addressed by a scientist in his or her public comments about risk can shape audience perceptions of the political orientations of the communicating scientist and thus his or her credibility, reflecting motivated reasoning to dismiss scientific evidence incongruent with participants' political beliefs [9,20,23].

Our results suggest when a scientist addresses the risks of controversial and politically divided scientific issues, members of the public are more likely to see the scientist as being a member of the political group associated with that issue, compared to when a scientist addresses a less controversial scientific issue. An important caveat is that perceptions of scientists' political orientations tended to cluster to the midpoint of the scales—indicating that, overall, Americans perceive scientists to be moderate in their political orientation. Communicating about a controversial issue context does not ‘brand’ a scientist as extremely politically conservative or liberal (or Republican or Democratic); however, it does incline perceptions that way.

This tilt was particularly noticeable when the scientist addressed the dangers of marijuana use (the ‘conservative’ issue), leading partisan audiences (both liberal and conservative) to rate the communicating scientist as more conservative and more Republican compared to other non-polarized issue contexts. This effect was more muted when the scientist addressed the risks of climate change. Of course, these results are inherently comparative—the marijuana issue context stands out because it often produced perceptions of the scientist as conservative or Republican, while the non-controversial comparison conditions (flu and severe weather) tended to produce perceptions of the scientist as slightly liberal or Democratic.

It is meaningful that a single exposure to a scientist providing risk information—absent any advocacy for specific solutions to reduce that risk—can shift these perceptions of political orientations depending only on the issue context. Therefore, this is a conservative test of the types of risk communication that scientists may engage in through their public comments. Previous research suggests more explicit kinds of advocacy—for example, recommending specific policy solutions to address scientific risks—may amplify attributions of political bias on the part of the communicating scientist compared to providing risk information alone, although these findings are mixed [55] and these effects may also depend on the ideological congruency of the policy [43].

An individual's political ideology also shaped their response to the risk communication efforts by the scientist in this study. We observe some disparity in how liberals versus conservatives used the issue context to inform their perceptions of the scientist's political orientations and translated those beliefs into credibility perceptions. Liberals demonstrated larger shifts than conservatives in their rating of the communicating scientist as more conservative and Republican when seeing risk communication about marijuana use versus severe weather. Meanwhile, the effects on perceptions of the scientist's political orientations when seeing the ‘liberal’ issue of climate change are stronger among conservative participants than liberals.

Moreover, the effects appear larger among conservative respondents when we consider the relationship between these perceptions of the scientist's political orientations and his credibility. Among conservatives, addressing the dangers of marijuana consistently improved credibility ratings via perceptions of the scientist's political beliefs, while a scientist addressing climate change risks tended to have lower credibility ratings among conservatives, at least compared to the flu issue context. Among liberals, however, we again see the reduction in credibility through perceptions of the scientist's political leanings for the issue of marijuana, but did not see a corresponding boost in credibility for the climate change issue. Several reasons may explain political asymmetry in the response to risk communication by scientists. First, perceptions of the scientific community as ‘liberal’ appear relatively well established compared to arguments that scientists are conservative—especially among political partisans on both ends of the continuum [40]. Therefore, when a scientist adopts a counter-stereotypical position and addresses a controversial science issue associated with conservatives, it may have more ability to influence attitudes rather than confirming existing views about the biases of scientists. Second, along these lines, our scientist appears to have been seen as slightly liberal and Democratic even in the non-controversial conditions, making it harder to perceive differences when the scientist engages in risk communication on the more explicitly ‘liberal’ issue of climate change. However, the analysis of moderates casts some doubt on these explanations, as the marijuana issue context did not appear to have a larger impact among this non-partisan group than the climate change context (see the electronic supplementary material, appendix).

This also raises a limitation of our study—while we attempted to select issues that differed in their relative controversy and political alignment, we can only examine a small number of the potential issues that scientists address as part of their public efforts. Additionally, the controversial issues examined—climate change and marijuana use—could exist in different domains of scientific communication—earth science and health science, respectively. It is possible that the stronger effects that we observed when a scientist addressed the marijuana issue occurred because it overlapped with a health, rather than earth science domain, where public acceptance of risk communication by scientists may be less controversial [56]. Likewise, we included two non-controversial ‘control’ conditions—severe weather and the flu—but occasionally saw differences between these control conditions in terms of ratings of the scientist's political orientations or credibility.^8^ Future research should continue to test how the features of an issue intersect with communication efforts by scientists to shape public perceptions of the issue itself as well as the scientific community, and how the effects might differ depending on the type of expert involved in the communications.

Our results could be interpreted as supporting a contextual model of issue context among liberals seeing a scientist engage in risk communication on a ‘conservative’ issue [9,20]. Further, it may be that any contextual effects of issue context are stronger among conservatives when considering different outcomes or when testing different issues [23], which our study cannot speak to. Our selection of climate change as a liberal issue is rooted in deep and long-standing divides between political groups in the United States, which may already inform perceptions of scientists and thus lose their effectiveness. A less salient scientific issue that engenders perceptions of a ‘liberal’ scientific establishment may be more successful in altering perceptions of the scientist's political orientations, especially among conservatives.

This study addresses an ongoing debate about the appropriate role for scientists in engaging in public commentary. For example, Kotcher *et al*. [57] suggested that different levels of scientific advocacy on the issue of climate change may not alter perceptions of the scientist's credibility, while van der Linden *et al*. [58] found that communicating the scientific consensus on climate change was effective in producing more accurate climate change beliefs among both Republicans and Democrats. These studies could be interpreted as supporting efforts for scientists to communicate publicly on the issue of climate change. However, both of those studies examine scientific communication within the issue of climate change, whereas the current study examines risk communication across a range of scientific issues. Our related work suggests that the effects of advocacy on perceptions of scientists can differ depending on the issue context in which the scientist is communicating [55], which may result from the differing perceptions of the political context surrounding the issues that this study investigates.

Moreover, Kotcher *et al*. [57] found that conservatives viewed the scientist who was communicating about climate change to be less credible than did liberals, regardless of the level of advocacy in the scientist's message. This current study suggests a mechanism for this effect—in choosing to talk about climate change, rather than another issue, the scientist provided a signal about his political beliefs, which was used to inform credibility perceptions.

Ultimately, we think the takeaway for scientists across these studies is that they must be cognizant of the potential consequences of engaging in public risk communication on contentious scientific issues, not that they need to avoid it entirely. This study suggests that when a scientist only speaks to the potential risks and consequences of an issue—avoiding any recommendation of specific policy solutions—the public can make assumptions about their political orientations based on this information. Even more problematic, after seeing a single scientist engaging in such communication, the public sometimes attributes these political labels to the scientific community more broadly, creating a potential reinforcing cycle of perceptions of scientists as political actors. Future research should test these links between assumptions about scientists' political orientations and other outcomes such as willingness to consider scientists' recommendations. Alternatively, it may be that scientists who explain their positive motivations for conducting research in such polarized issue contexts (e.g. serving the public good [55]) may negate the effects found here, a possibility that future research should examine. Furthermore, reframing the issue in a way that resonates with an audience's political views could help attenuate the attributions some individuals might make about scientists based solely on the issue under consideration [59]. Scientists should be aware that audience members may make inferences about the communicating scientist's political orientations and their credibility when they engage in risk communication about controversial issues.

## Supplementary Material

Perceptions of Scientist Appendix

## Supplementary Material

Polarization_ROS_RR.sav

## Supplementary Material

Perceptions of scientists final syntax

## Supplementary Material

H2 output.spv

## Supplementary Material

H3 output.spv

## Supplementary Material

H4 output.spv

## Supplementary Material

PLoS ONE ms

## Footnotes

1 *USA Today* was selected to limit the potential for partisan perceptions of the news to influence perceptions of Dr Wilson. While choosing a news outlet entirely absent from partisan perceptions is difficult, *USA Today* tends to be read roughly equally by Democrats, Republicans and Independents [1], and tends to be more trusted than distrusted by all groups except consistent conservatives, who are somewhat more likely to distrust than trust *USA Today* [10,47]. Moreover, as this source is consistent across conditions, it should not influence the proposed relationships between issue context and participant political ideology.

2 These conditions are extracted from a larger experimental design, wherein we also manipulated whether the scientist offered a non-controversial or a controversial policy solution for each of the four issues examined here. In this paper, we are interested in the effects of *issue context* in risk communication and its relationship to perceptions of scientific political orientations and credibility, rather than the effects of scientific advocacy, which we believe is a different but related question. Analyses from a paper explicitly exploring scientific advocacy are reported elsewhere.

3 Our original design divided the four issues using a 2 (polarizing scientific issue: controversial versus non-controversial) × 2 (issue domain: health versus earth science), and counter-balanced the ‘lean’ of the polarized issues (one conservative and one liberal). However, with this analysis, perceptions of scientists' political leaning are hypothesized to depend on the partisan direction—and thus, separating the two polarized issues from one another is necessary. In this analysis, we do not examine differences between the earth and health science contexts.

4 See http://success.qualtrics.com/rs/qualtrics/images/ESOMAR%2028%202014.pdf for a description of Qualtrics and their research services.

5 An additional 1645 participants were in the advocacy conditions reported elsewhere, for a total of 2453 participants in the full sample. Participants who failed two of three attention checks were also excluded from the final sample. See the electronic supplementary material, appendix for exact question wording. All statistics are reported only for participants in the four experimental conditions examined in this paper.

6 We feel it is justified to treat ideology as a categorical, rather than continuous variable, because as discussed in the Introduction, while there are clear expectations for liberals and conservatives, there are not for moderates, necessitating analysis by ideological categories, rather than linearly. Furthermore, the relationship between participant ideology and perception of scientists' political leanings was not always linear.

7 We performed additional analyses testing whether Dr Wilson's issue context for public communication impacted perceptions of scientists' political orientations and their credibility. These analyses suggest that while there were some effects of issue context on perceptions of scientists' party affiliation overall in a consistent direction as evaluations of Dr Wilson, they did not generally lead to indirect effects of issue context on the trustworthiness of the scientific community, either alone or in conjunction with participants' political ideology. Instead, we observe a direct effect of issue context on perceptions of scientists' trustworthiness, with a scientist who addresses *either* controversial issue of marijuana or climate change directly leading to lower ratings of the trustworthiness of the scientific community compared to the flu issue (but not severe weather). We report these analyses in the electronic supplementary material, appendix.

8 Specifically, the flu issue context produced ratings of Dr Wilson as significantly (*B* = −0.31, s.e. = 0.14, *p* = 0.03) more conservative than the severe weather issue context, and marginally more credible (*B* = 0.25, s.e. = 0.13, *p* = 0.07).

## Data accessibility

The data are included as a supplementary file to this publication.
