# Supplementary material for: Scientific risk communication about controversial issues influences public perceptions of scientists' political orientations and credibility
Source: R Soc Open Sci. 2018 Feb 21;5(2):170505. doi: 10.1098/rsos.170505 (PMC5830709; doi:10.1098/rsos.170505)

**Appendix 1: Experimental Conditions**

**Flu Issue Context**

Dr. Dave Wilson, a recognized international expert in the field of public health, recently published an op-ed in *USA Today*. In the article, he said:

“Evidence from recent scientific studies reveal that the flu is even more dangerous and costly than once believed. The flu contributes to pneumonia, bronchitis, fatigue, and even premature death. Moreover, because so many people are affected by the flu, it is increasing everyone’s insurance costs.

**Marijuana Issue Context**

Dr. Dave Wilson, a recognized international expert in the field of public health, recently published an op-ed in *USA Today*. In the article, he said:

“Evidence from recent scientific studies reveal that marijuana use is even more dangerous and costly than once believed. Marijuana use contributes to mental health problems, including depression and anxiety disorders, and it is addictive for many users. Moreover, because so many people are affected by marijuana use, it is increasing everyone’s insurance costs.

**Severe Weather Issue Context**

Dr. Dave Wilson, a recognized international expert in the field of meteorology, recently published an op-ed in *USA Today*. In the article, he said:

“Evidence from recent scientific studies reveals that severe weather is more dangerous and costly than once believed. Severe weather (such as floods and hurricanes) contributes to injuries, deaths and property damage, and has increased markedly in recent years. Moreover, because so many people are affected by severe weather, it is increasing everyone’s insurance costs.

**Climate Change Issue Context**

Dr. Dave Wilson, a recognized international expert in the field of meteorology, recently published an op-ed in *USA Today*. In the article, he said:

“Evidence from recent scientific studies reveal that climate change is more dangerous and costly than once believed. Carbon dioxide contributes to damage to human health, food shortages, species loss, and economic impacts on the order of 5% of global GDP. Moreover, because so many people are affected by climate change, it is increasing everyone’s insurance costs.

**Appendix 2: Attention Checks**

*Attention Check #1, N=*1089 failed

The following statement was embedded and randomly rotated with five other Likert-style questions, with response options ranging on a seven-point scale from “Strongly disagree” to “strongly agree.”

If you are paying attention, please select “somewhat disagree” for this statement.

*Attention Check #2, N=*1880 failed

On a separate page, participants viewed the following question.

Now we would like to get a sense of your general preferences.

Most modern theories of decision making recognize that decisions do not take place in a vacuum. Individual preferences and knowledge, along with situational variables can greatly impact the decision process. To demonstrate that you’ve read this much, just go ahead and select both red and green among the alternatives below, no matter what your favorite color is. Yes, ignore the question below and select both of those options.

What is your favorite color? [White, Black, Red, Pink, Green, Blue]

*Attention Check #3, N=*1938 failed

On a separate page, participants viewed the following question.

People are very busy these days and many do not have time to follow what goes on in the government. Some do pay attention to politics but do not read questions carefully. To show that you’ve read this much, please ignore your real answer to the question below and instead select both "extremely interested" and "not interested at all". That’s right, select two choices, both "extremely interested" and "not interested at all".

How interested are you in information about what’s going on in government and politics?

[Extremely interested, very interested, moderately interested, slightly interested, not interested at all]

| *Sample Exclusions* | *N* |
| --- | --- |
| Failed 2 or more attention checks | 1,667 |
| Did not complete survey | 791 |
| Speeder (spent less than 4 minutes, 20 seconds on survey) | 40 |
| Did not give consent | 81 |
| Over quota | 3,932 |
| **Total Removed** | **6,511** |

**Appendix 3: Supplemental Analyses for Perceptions of Scientific Community**

- **Table A3.1.** Coefficients predicting Scientists’ Political Orientation from Issue Context

|  |  | - Scientists’ Ideology | - Scientists’ Party | |
| --- | --- | --- | --- | --- |
| - Intercept | | - 0.379*** | - 0.621*** | |
| - Marijuana | |  |  | |
|  | - vs. Flu | - -0.065 | - -0.209 | |
|  | - vs. Severe Weather | - 0.045 | - -0.329* | |
| Climate Change | |  |  | |
|  | - vs. Flu | - 0.016 | - 0.265* | |
|  | - vs. Severe Weather | - 0.126 | - 0.145 | |
|  | |  |  | |
| - Liberal (vs. Conservative) | | - -0.052 | - -0.621*** |  |
| - Moderate (vs. Conservative) | | - -0.270** | - -0.191*** |  |
| - Note: Entries are unstandardized regression coefficients. Each model was run twice times to generate all comparisons: once with the reference category of severe weather and again with the reference category of flu. The intercept is from the model with severe weather as the reference category. In these models, a higher number indicates a more liberal or Democratic orientation. - ****p* < .001, ** *p* < .01, * *p* < .05, + *p* < .10 | | | | |

**Table A3.2:** Testing Mediation of Issue Context on Perceptions of Trustworthiness for the Scientific Community, through Political Orientations of Scientists for All Participants

|  |  |  | Scientists’ Political Orientation |  | Credibility | |
| --- | --- | --- | --- | --- | --- | --- |
|  |  |  |  |  | *Direct Effect* | *Indirect Effect through Political Orientation* |
| Political Orientation:  Scientists’ Ideology | |  |  |  |  |  |
|  | Marijuana vs. Flu |  | -0.09 |  | -0.25* | 0.01 |
|  | CC vs. Flu |  | 0.02 |  | -0.29* | -0.00 |
|  | Marijuana vs. SW |  | .02 |  | 0.07 | -0.00 |
|  | CC vs. SW |  | 0.13 |  | 0.04 | -0.01 |
|  | Scientists’ Ideology |  |  |  | -0.10* |  |
| Political Orientation:  Scientists’ Party | |  |  |  | | |
|  | Marijuana vs. Flu |  | -0.23+ |  | -0.26* | 0.02 |
|  | CC vs. Flu |  | 0.30* |  | -0.26* | -0.03 |
|  | Marijuana vs. SW |  | -.37** |  | 0.04 | 0.03 |
|  | CC vs. SW |  | 0.17 |  | 0.04 | -0.01 |
|  | Scientists’ Party |  |  |  | -0.08* |  |

Note: Entries are the point estimate for the indicated relationship. In these models, a higher number for perceptions of scientists’ political orientation indicates a more liberal or Democratic orientation. ** *p* < .01, * *p* < .05, + *p* < .10.

**Table A3.3:** Summary of Results for Magnitude of Differences in the Effect of Condition, between Liberals and Conservatives

|  |  | Scientists’ Ideology^ | | | Scientists’ Party^†^ | | | |  |  |
| --- | --- | --- | --- | --- | --- | --- | --- | --- | --- | --- |
|  |  | Liberal | Conservative | Diff. | | Liberal | Conservative | Diff. | |  |
| Climate Change | |  |  |  | |  |  |  | |  |
|  | vs. Flu | 0.062 | -0.108 | ns | | 0.348 | -0.228 | ns | |  |
|  | vs. Severe Weather | 0.110 | 0.067 | ns | | 0.022 | 0.026 | ns | |  |
| Marijuana | |  |  |  | |  |  |  | |  |
|  | vs. Flu | -0.043 | -0.213 | ns | | -0.335 | -0.492+ | ns | |  |
|  | vs. Severe Weather | 0.005 | -0.038 | ns | | -0.661* | -0.238 | ns | |  |
| Note: Entries in the Liberal and Conservative columns are the unstandardized regression coefficients generated from the PROCESS macro (Hayes, 2013); they represent the simple effect of the issue context comparison for the ideology noted. Each model was run twice to generate comparisons: once with the reference category of flu and again with severe weather. The “Diff.” column is the significance of the difference in the coefficients, as measured by the interaction term between the comparison and liberal vs. conservative ideology; significant differences in the “Diff” column indicate that the difference in magnitude between the liberal and conservative coefficients for the comparison is not likely due to chance.  ^ Liberal was coded high for political ideology. Therefore, positive coefficients indicate a more liberal score and negative coefficients indicate a more conservative score.  † Democrat was coded high for political party. Therefore, positive coefficients indicate a more Democratic score and negative coefficients indicate a more Republican score. | | | | | | | | | | |

****p* < .001, ** *p* < .01, * *p* < .05, + *p* < .10

**Table A3.4.** Testing the Moderated Mediation of Topic on Trust in the Scientific Community, through Political Orientation of Scientists’, as Mediated by Participant’s Political Ideology

|  |  | **Scientists’ Political Orientation** | **Credibility** | | |
| --- | --- | --- | --- | --- | --- |
|  |  |  |  | Conditional Indirect Effects | |
|  |  | Issue X Participant Ideology | Political Orientation X Participant Ideology | Conservative | Liberal |
| Scientists’ Ideology | |  |  |  |  |
|  | Marijuana vs. Flu | -.17 | .78*** | .10 | -.01 |
|  | Climate change vs. Flu | .17 |  | .05 | .02 |
|  | Marijuana vs. Severe Weather | .04 |  | .02 | .00 |
|  | Climate Change vs. Severe Weather | .04 |  | -.03 | .04 |
| Scientists’ Party | |  |  |  |  |
|  | Marijuana vs. Flu | .16 | .38*** | .14 | -.04 |
|  | Climate change vs. Flu | .58 |  | .06 | .03 |
|  | Marijuana vs. Severe Weather | -.42 |  | .07 | -.07* |
|  | Climate Change vs. Severe Weather | -.00 |  | -.01 | .00 |

**Note:** ****p* < .001, ** *p* < .01, * *p* < .05.

**Appendix 4: Supplemental Analyses for Moderates Only**

**Table A4.1.** Coefficients Predicting Dr. Wilson’s Political Orientation from Issue Context, for Moderates only

|  |  | Dr. Wilson Ideology | Dr. Wilson Party |
| --- | --- | --- | --- |
| Intercept | | 0.136 | 0.136 |
| Marijuana | |  |  |
|  | vs. Flu | -0.136 | -0.341+ |
|  | vs. Severe Weather | -0.145 | -0.755** |
| Climate Change | |  |  |
|  | vs. Flu | 0.255* | 0.686** |
|  | vs. Severe Weather | 0.246+ | 0.272 |
| Note: Entries are unstandardized regression coefficients. Each model was run 3 times to generate all comparisons: once with the reference category of severe weather, and again with the reference category of flu, and marijuana. The intercept is from the model with severe weather as the reference category.  ****p* < .001, ** *p* < .01, * *p* < .05, + *p* < .10 | | | |

**Table A4.2:** Simple Mediation for Perceptions of Dr. Wilson’s Political Orientations and Credibility Among Moderates Only

|  |  |  | Dr. Wilson’s Political orientation |  | Credibility | |
| --- | --- | --- | --- | --- | --- | --- |
|  |  |  |  |  | *Direct Effect* | *Indirect Effect through Political Orientation* |
| Political Orientation:  Dr. Wilson’s Ideology | |  |  |  |  |  |
|  | Marijuana vs. Flu |  | -0.14 |  | -0.70** | -0.01 |
|  | CC vs. Flu |  | 0.26* |  | -0.55** | 0.02 |
|  | Marijuana vs. SW |  | -0.15 |  | 0.06 | -0.01 |
|  | CC vs. SW |  | 0.25+ |  | 0.10 | 0.02 |
|  | Dr. Wilson’s Ideology |  |  |  | 0.09 |  |
| Political Orientation:  Dr. Wilson’s Party | |  |  |  | | |
|  | Marijuana vs. Flu |  | -0.34+ |  | -0.68*** | 00.03 |
|  | CC vs. Flu |  | 0.69** |  | -0.58** | 0.06 |
|  | Marijuana vs. SW |  | -0.75** |  | -0.01 | -0.06 |
|  | CC vs. SW |  | 0.27 |  | 0.10 | 0.02 |
|  | Dr. Wilson’s Party |  |  |  | 0.08 |  |

Note: Entries are the point estimate for the indicated relationship. In these models, a higher number for perceptions of Dr. Wilson’s political orientation indicates a more liberal or Democratic orientation. ****p* < .001, ** *p* < .01, * *p* < .05, + *p* < .10.

**Appendix 5: Question Wording and Visual for Perceptions of Dr. Wilson’s Credibility**

Please indicate your impression of Dr. Wilson by choosing the appropriate number between the pairs of adjectives below. The closer then number is to either adjective, the more certain you are of your evaluation.

Not at all expert 1 – 2 – 3 – 4 – 5 – 6 – 7 – 8 Extremely expert

Extremely sincere 1 – 2 – 3 – 4 – 5 – 6 – 7 – 8 Not at all sincere

Not at all sensitive 1 – 2 – 3 – 4 – 5 – 6 – 7 – 8 Extremely sensitive

Not at all competent 1 – 2 – 3 – 4 – 5 – 6 – 7 – 8 Extremely competent

Not at all trustworthy 1 – 2 – 3 – 4 – 5 – 6 – 7 – 8 Extremely trustworthy

Is concerned about society a great deal 1 – 2 – 3 – 4 – 5 – 6 – 7 – 8 Isn’t concerned about society a great deal

Not at all intelligent 1 – 2 – 3 – 4 – 5 – 6 – 7 – 8 Intelligent

Not at all honest 1 – 2 – 3 – 4 – 5 – 6 – 7 – 8 Extremely honest

Note: Although this is a 7-point scale, participants were able to select one of eight radio buttons. We have acknowledged this issue in the methods section of the main document.


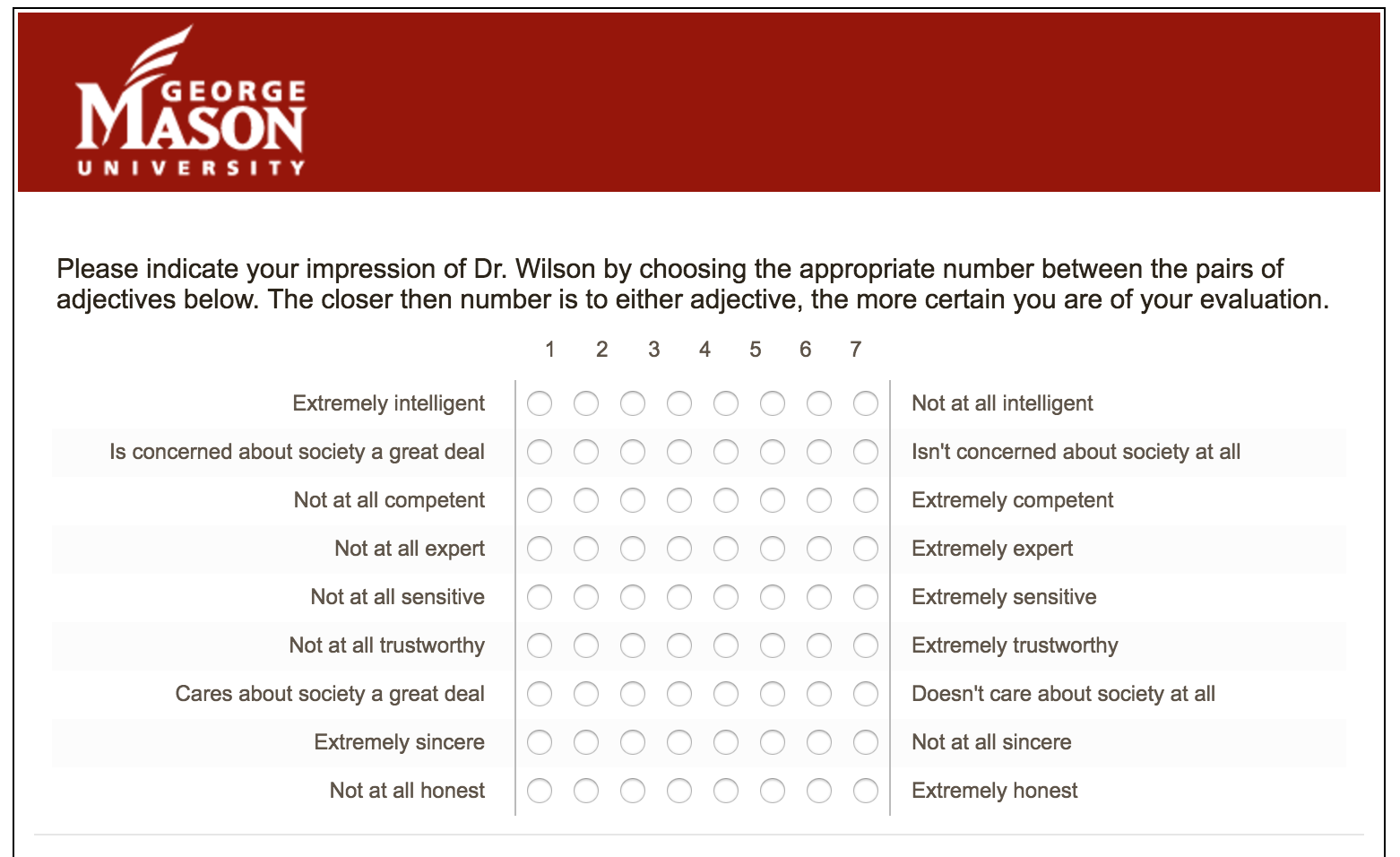

Supplement: Perceptions of Scientist Appendix [file rsos170505supp1.docx]
