# Supplementary material for: Scientific risk communication about controversial issues influences public perceptions of scientists' political orientations and credibility
Source: R Soc Open Sci. 2018 Feb 21;5(2):170505. doi: 10.1098/rsos.170505 (PMC5830709; doi:10.1098/rsos.170505)
Supplement: PLoS ONE ms [file rsos170505supp7.pdf]

PLOS | ONE

RESEARCH ARTICLE

Controversy matters: Impacts of topic and solution controversy on the perceived credibility of a scientist who advocates

Lindsey Beall*, Teresa A. Myers, John E. Kotcher, Emily K. Vraga, Edward W. Maibach

Department of Communication, George Mason University, Fairfax, Virginia, United States of America

* LBeall3@gmu.edu

Check for updates

OPEN ACCESS

Citation: Beall L, Myers TA, Kotcher JE, Vraga EK, Maibach EW (2017) Controversy matters: Impacts of topic and solution controversy on the perceived credibility of a scientist who advocates. PLoS ONE 12(11): e0187511. https://doi.org/10.1371/journal.pone.0187511

Editor: James Wilsdon, University of Sheffield, UNITED KINGDOM

Received: April 8, 2017

Accepted: October 17, 2017

Published: November 14, 2017

Copyright: © 2017 Beall et al. This is an open access article distributed under the terms of the Creative Commons Attribution License, which permits unrestricted use, distribution, and reproduction in any medium, provided the original author and source are credited.

Data Availability Statement: All data files are available from the Zenodo dataset repository (doi: 10.5281/zenodo.495653).

Funding: This research was supported by the National Aeronautics and Space Association (NASA.gov) [Award #: NNX11AQ80G]. The funders had no role in study design, data collection and analysis, decision to publish, or preparation of the manuscript.

Competing interests: The authors have declared that no competing interests exist.

Abstract

In this article, we focus on the potential influence of a scientist's advocacy position on the public's perceived credibility of scientists as a whole. Further, we examine how the scientist's solution position (information only, non-controversial, and controversial) affects the public's perception of the scientist's motivation for sharing information about specific issues (flu, marijuana, climate change, severe weather). Finally, we assess how perceived motivations mediate the relationship between solution position and credibility. Using data from a quota sample of American adults obtained by Qualtrics (n = 2,453), we found that in some conditions advocating for a solution positively predicted credibility, while in one condition, it negatively predicted scientist credibility. We also found that the influence of solution position on perceived credibility was mediated by several motivation perceptions; most notably through perception that the scientist was *motivated to: (a) serve the public and (b) persuade the public*. Further results and implications are discussed.

Introduction

Scientists can play various roles in policy decision-making processes, but the appropriateness of those roles is debated among researchers and scientists themselves. Some scientists see it as their responsibility to interpret scientific information and to advocate for specific policies [1, 2]. Other scientists avoid engaging in advocacy, citing past negative experiences with public scrutiny, or concerns about the detrimental effects on their or the scientific community's scientific credibility [1–4]. The strongly held feelings on both sides of this issue are informed more by assumptions and anecdote than by evidence, as relatively little research has been done on the impact of advocacy by scientists on public trust.

The definition of advocacy itself remains open to debate. Although some scholars argue that advocacy is binary–a scientist is either engaging in advocacy or not [4]–others conceptualize advocacy as multi-categorical (e.g., Roger Pielke's framework) [5] or continuous (Simon Donner's science-advocacy continuum) [6]. Donner's continuum is based on the extent of normative judgment inherent in a message—with a "scientific" end of the continuum being more objective and an "advocacy" end that is more subjective. For these scholars, scientists'

role in the decision-making process exists on a spectrum, ranging from “pure scientists,” who provide their research as objective facts about an issue, to “honest brokers,” who address a range of potential policy solutions, to finally “issue advocates,” who encourage decision-making and solutions for specific issues. In other words, sharing information about a recent scientific finding, is a lesser form of advocacy than suggesting that specific actions that need to be taken.

In the present study, our interest is in comparing responses to two types of policy advocacy—favoring a controversial or a non-controversial policy solution—against a purely informational statement. Here, we consider a policy solution to be controversial if it is likely to provoke polarized attitudes among respondents due to ideologically motivated dissonance. Conversely, we consider a policy solution to be non-controversial if it is unlikely to provoke dissonance and instead should enjoy relatively widespread support across ideological groups. Nisbet, Cooper, and Garret [7] found that participants’ trust in scientists wavered when they were presented with ideologically dissonant information, regardless of political orientation. When both conservatives and liberals were presented with science communication information that challenged their pre-held beliefs, they reacted negatively. Therefore, in our study, a perceived controversial solution refers to a polarizing solution, while the non-controversial solution still implies a normative judgment (advocacy), but a judgment that many people agree is broadly good for society.

The debate of whether scientists have a responsibility–or even the latitude–to advocate for policy solutions is fierce among scientists and science communication researchers. The most common argument against advocacy is that it can negatively influence credibility [8]. Renn and Levine [9] and Kasperson, Golding, and Tuler [10] both determined that four key dimensions of credibility in risk communication contexts are perceptions of commitment, competence, caring, and predictability. Further, Peters, Covello, & McCallum [11] adapted the determinants of credibility in risk communication to include perceptions of knowledge and expertise, openness and honesty, and care and concern. It is often assumed that higher levels of advocacy could result in lower perceived credibility of the scientist [6]. Advocating for specific solutions, especially ones deemed controversial could potentially be risky for scientists. Recent work, however, has found that advocacy does not always result in decreased credibility [12]. In the current research, we add to the empirical discussion of the effect of scientist’s advocacy by testing the impact of advocacy for controversial and non-controversial solutions on the credibility of scientists. For example, a scientist advocating for a relatively non-controversial, or an ideologically congruent, solution to a public health issue–such as washing one’s hands frequently to prevent the spread of the flu–will likely face a less skeptical public than scientists proposing a controversial solution, such as a carbon tax, in order to mitigate climate change. We also use a range of different scientific topics (i.e., flu, marijuana, severe weather, and climate change) to compare the effects of advocacy positions on scientist credibility in numerous contexts. Specifically, we propose the following research questions.

RQ1a: What effect will advocating for a *non-controversial* solution have on the perceived credibility of the communicating scientist, compared to an informational statement solely about the risks associated with an issue?

RQ1b: What effect will advocating for a *controversial* solution have on the perceived credibility of the communicating scientist, compared to an informational statement solely about the risks associated with an issue?

The effects of perceived motives on credibility

Of course, scientific advocacy may not only directly impact perceptions of scientific credibility, but also shape the perceived motives of the scientists engaging in this type of communication.

The theoretical role of perceived motives in shaping more general perceptions of scientists has its roots in attribution research [13]. Research on attribution has shown that people are likely to make inferences about the reasons an individual (or members of a group) engage in a particular behavior [14]. Those inferences are then used to form judgments about more stable characteristics associated with an individual or group [15, 16]. Research in risk communication, specifically, has identified the attribution of certain values or motives to an agent as a key factor involved in the formation of trust perceptions [17].

When it comes to evaluating scientific information, individuals are likely to assess why the scientist is sharing information. An experiment about scientists' advocacy found that the public perceived that the scientist featured in the experiment intended to persuade the public–both when the scientist only provided information about recent findings about climate change, *and* when the scientist was advocating for a solution (although the perceived intention to persuade was higher in conditions were the scientist was advocating for a solution) [17]. Based on these findings, it is apparent that the level of advocacy, can influence perceived motivations. We build on this research to incorporate controversy of the solution into the model, thus testing whether not only the level of advocacy but also the level of the controversy of the solution, influences perceived motivations. As incorporating controversy into the advocacy model has not been widely researched, the following research question is proposed.

RQ2: Will advocacy position (information only, non-controversial solution, controversial solution) be related to perceived motivations (discussed below) of scientists?

Relatively little research has examined the effect of perceived motives on scientists' credibility. In an exploratory study using a small convenience sample, Fiske & Dupree (2014) asked respondents to list reasons to trust climate scientists and reasons to distrust climate scientists. Consistent with past research cited above, their participants attributed self-serving or political motives such as trying to lie with statistics, gain research money, and pursue a liberal agenda as reasons to distrust climate scientists [18]. Furthermore, the more a communicator's advocated position is attributed to their personal political views, they more they are seen as biased, and the more their position is attributed to factual evidence, the less they are seen as biased [19]. Alternatively, respondents attributed more altruistic motives–such as a desire to educate the public, save humanity, and save the environment–as reasons to trust climate scientists [18]. This accords with prior research by Critchley [20] who found that university funded scientists were perceived to be more benevolent and their research was more beneficial to the public than privately funded scientists, who were perceived to be more interested in personal gain. Overall, public trust was greater for university funded scientists than private scientists. These findings are consistent with a well-established finding about persuasion: when communicators are believed to be acting in their own self-interest or in service of special interests, they are less likely to be trusted, whereas belief that communicators are acting *against* their own self-interest can increase trust and goodwill [21, 22]

Rabinovich, Morton, and Birney [23] tested the effects of two different perceived motives on trust in scientists: belief that climate scientists are motived to inform the public about the impacts of climate change versus to persuade the public to take a particular course of action on the issue. They found that a purely informative message led to higher levels of trust when it was consistent with a pre-existing belief that most climate scientists have informative goals, and it led to lower levels of trust when the audience was led to believe scientists had persuasive goals. Conversely, a persuasive message had a positive effect on trust when the audience was led to believe most climate scientists have persuasive intentions, and it had a negative effect on trust when the audience believed most climate scientists have informative intentions. This finding is at odds with a common assumption among opponents to advocacy by scientists, which is that perceived persuasive goals will invariably erode scientists' credibility [4, 24].

In our study, we examine a series of motives that may influence perceived credibility of the communicating scientist. We test whether participants believe the scientist in the experiment is motivated by: a desire to provide scientific evidence, a desire to inform the public, a desire to serve the public, a desire to persuade the public, personal promotion, and one's political views. Most of these motivations—specifically those benefiting the public versus those benefiting the scientist—have a positive and negative valence, respectively. Is it less clear, however, if the motive to persuade the public has a valence. Thus, the following hypotheses and research question are proposed.

H1: Belief that the communicating scientist's statement was motivated by a desire to a) provide scientific evidence, b) inform the public, and c) serve the public will be positively associated with perceived credibility.

RQ3: How will belief that the communicating scientist's statement was motivated by a desire to persuade the public relate to credibility?

H2: Belief that the communicating scientist's statement was motivated by a desire to a) gain personal promotion, and b) promote their personal political views will be negatively associated with perceived credibility.

RQ4: Do perceived motives mediate the relationship between position advocacy and perceived credibility?

Method

Sample

In October and November of 2015 we surveyed a sample of 2,453 adult Americans. Approval was obtained from the Institutional Review Board. Participants provided consent to participate in the study through a question at the beginning of the survey. Participants were members of an online panel maintained by Qualtrics and were quota sampled to match the US population on gender, age, and education. Participants ranged in age from 18–87, with an average age of 46 years old. 48% of participants were male (52% female); 11% of participants had less than a high school education, 31% had a high school education, 30% had some college, 18% were college graduates, and 10% had a post-graduate education.

Attention Checks. Three attention check items were used test participant attention; those who failed any two attention checks were removed from the survey and did not count toward our total of 2,453 participants.

Procedure

Participants were randomly assigned into one of twelve conditions in a 4 (Topic: Flu, Marijuana, Severe weather, Climate change) x 3 (Solution Position: Information only, Non-controversial solution, Controversial solution) factorial design. In each condition, participants read a USA Today op-ed by a scientist named Dr. Wilson. The information only op-ed contained scientific information about the dangers of the flu, marijuana, climate change, or severe weather. The non-controversial solution position included the scientific information and a suggestion to implement legislation that warns the public of those dangers. The controversial solution position included the scientific information and a suggestion to introduce legislation that will regulate and mandate effective solutions. Full stimulus material is provided in S1 Appendix, see Table 1 for an example. After reading their assigned op-ed, participants answered a series of survey questions.

Results of a pre-test validated the operationalization of information only, non-controversial, and controversial solutions. Participants from a Qualtrics quota sample, matched to national characteristics, (N = 202) reported the manipulated controversial solutions to be significantly

Table 1. Sample experimental stimuli.

	Flu
Information only	Dr. Dave Wilson, a recognized international expert in the field of public health, recently published an op-ed in USA Today. In the article, he said: "Evidence from recent scientific studies reveal that the flu is even more dangerous and costly than once believed. The flu contributes to pneumonia, bronchitis, fatigue, and even premature death. Moreover, since so many people are affected by the flu, it is increasing everyone's insurance costs."
Non-controversial	[All of the content from the information only condition, plus:] The flu threatens everyone and action should be urgently taken to reduce the risk. One effective approach for addressing the problem of the flu is to **introduce legislation to post information signs about the link between hand washing and the flu in all restaurants and public buildings**. My research, and research conducted by many other experts, suggests that this simple and inexpensive action is an effective way to protect Americans from the risks of the flu."
Controversial	[All of the content from the information only condition, plus:] The flu threatens everyone and action should be urgently taken to reduce the risk. One effective approach for addressing the problem of the flu is to **introduce legislation to require all Americans to get flu shots every year**. My research, and research conducted by many other experts, suggests that this simple and inexpensive action is an effective way to protect Americans from the risks of the flu."

Note: The bolded sections indicate the difference between the non-controversial and controversial solutions. The information was not bolded in the actual stimulus material shown to participants.

https://doi.org/10.1371/journal.pone.0187511.t001

more controversial than the non-controversial solutions for each issue domain. Within each issue, participants were randomly assigned to see either the non-controversial or controversial solution first. Results of a series of independent samples *t*-tests comparing the means of the two groups (i.e., participants who saw the non-controversial solution first and participants who saw the controversial solution first) indicated that the controversial solutions were seen as more controversial than the non-controversial solutions at $p < .001$ for all four issues. (Means of reported controversy -measured on a five-point scale- for the two solutions, by topic: Flu: $M_{Controversial} = 4.10$, SD = 2.07 $M_{Non\text{-}controversial} = 1.81$, SD = 1.09; Climate change: $M_{Controversial} = 3.82$, SD = 1.09, $M_{Non\text{-}controversial} = 2.43$, SD = 1.21; Severe weather: $M_{Controversial} = 3.17$, SD = 1.09, $M_{Non\text{-}controversial} = 1.83$, SD = 1.05; Marijuana: $M_{Controversial} = 3.50$, SD = 1.07, $M_{Non\text{-}controversial} = 2.25$; SD = 1.13.) Additionally, after collecting the data reported in this paper, we conducted independent samples *t*-tests for each issue to assess whether the study participants assigned to the non-controversial and controversial conditions recognized the manipulation. Participants were asked to report on the controversy of the solution. Results suggest that the participants' responses mirrored the condition they were assigned. Means of participants exposed to the controversial op-ed were significantly higher than the means of the participants exposed to the non-controversial op-ed. Results were significant across issues at the .001 level. Flu: $M_{Controversial} = 3.64$, SD = 1.14, $M_{Non\text{-}controversial} = 1.96$, SD = 1.21; Climate change: $M_{Controversial} = 3.51$, SD = 1.04, $M_{Non\text{-}controversial} = 2.84$, SD = 1.07; Severe weather: $M_{Controversial} = 3.20$, SD = 1.16, $M_{Non\text{-}controversial} = 2.36$, SD = 1.23; Marijuana: $M_{Controversial} = 3.25$, SD = 1.15 $M_{Non\text{-}controversial} = 2.77$, SD = 1.19).

Measures

Credibility. Credibility was measured with nine items adapted from McCroskey and Teven (1999) on a 8-point scale that assessed the scientist's expertise, intelligence, competence, trustworthiness, sensitivity, sincerity, concern for society, care for society, and honesty [25]. For each of these components, participants were presented with a semantic differential task,

with "not at all [characteristic, e.g., intelligent]" on one side and "extremely [characteristic, e.g., intelligent]" on the other, and asked to choose the radio between the pairs that appropriately matched with their beliefs, with instructions that the closer the radio button was to the corresponding label, the more certain they were indicating their evaluation was. These were coded such that 1 indicated not at all and 8 meant extremely, and averaged across all items (M = 5.77, SD = 1.42). Reliability of this scale was sufficient ($\alpha = .88$). Unfortunately, after conducting the experiment, we noticed a slight error in the programming of the measurement of credibility. The credibility measure included in the questionnaire had eight radio buttons from which the participant could choose, but was labeled from 1–7 (the labels appeared in-between the radio buttons). See S1 Appendix for a visual representation of this problem, and the discussion section for our thoughts on the implications of this programming error.

Perceived motives. Participants were asked to evaluate Dr. Wilson's motivation for writing his op-ed. They were provided with six questions, each asking about a different motive: to provide impartial information (M = 4.59, SD = 1.82), his scientific evaluation of the evidence (M = 5.31, SD = 1.48), his desire to serve the public (M = 5.37, SD = 1.43), to persuade people to take action (M = 5.57, SD = 1.40), his desire for personal promotion (M = 3.58, SD = 1.85), and his political views (M = 4.02, SD = 1.82). Full wording is available in the supplemental material. They were asked to respond on a 7-point Likert scale from strongly disagree (coded 1) to strongly agree (coded 7).

Statistical analysis

We conducted regression analyses for our first hypothesis and first three research questions, dummy coding solution position (reference categories are noted in the discussion of the results). We utilized the PROCESS macro, version 2.15 [26] to conduct our mediation analyses. We used one model (including all motivations in parallel) for each of the four topics. Furthermore, we ran collinearity diagnostics and did not find evidence of collinearity among the perceived motives.

Results

To examine how the controversy level of solutions influences credibility (RQ1a&b), we predicted credibility from solution position, with the information-only condition as the reference category (see Table 2 for all coefficients). Advocating for a controversial solution had a significant negative impact on credibility, compared to the information-only condition, in the flu topic only; alternatively, it had a significant positive effect on credibility in the severe weather topic. Specifically, those who saw the controversial solution for the flu topic indicated a credibility score .38 units lower than those who saw the information only condition; conversely, those who saw the controversial solution for the severe weather topic indicated a credibility score .26 units higher than those who saw the information only condition. The non-controversial solution condition produced significantly higher credibility ratings for the communicating scientist than the information-only condition for 2 out of 4 topics (severe weather and climate change). In other words, scientist credibility was .28 units higher for participants who saw the severe weather non-controversial solution compared to participants who saw the information only op-ed. Similarly, scientist credibility was .29 units higher for participants who saw the climate change non-controversial solution for compared to participants who saw the information only op-ed.

To address RQ2, we examined whether the non-controversial and controversial solutions had an effect on the scientist's perceived motivations, relative to an information-only statement (see Table 3 for all results). Both solution types (controversial and non-controversial)

Table 2. Total effects of non-controversial and controversial solutions on credibility, in comparison to the information only condition.

	Credibility
Controversial vs. Information Only	
Flu	-0.38***
Marijuana	0.17
Severe Weather	0.26*
Climate Change	0.01
Non-Controversial vs. Information Only	
Flu	0.22
Marijuana	-0.05
Severe Weather	0.28*
Climate Change	0.29*

Note: Entries are unstandardized regression coefficients and can be interpreted as the difference in the means between the two conditions.

* $p < .05$,

*** $p < .001$

https://doi.org/10.1371/journal.pone.0187511.t002

were significantly higher than the information only condition in the perceived motivation of *persuading the public*, across all four topics. In order words, the perception that the scientist wanted to persuade the public to take action was significantly higher for participants who read either the controversial or non-controversial, compared to participants who read the information-only statements.

Relative to the information only condition, the non-controversial solution resulted in significantly higher perceptions that the scientist's aim was to *serve the public* for 3 out of 4 topics (marijuana was the exception). The results were more mixed for the controversial solutions. In one case (flu), the controversial solution resulted in lower perception that the scientist desired to serve the public; in another case (severe weather) the controversial solution resulted in higher perception that the scientist desired to serve the public.

For the motivation of *informing the public*, there were no observed differences between the non-controversial solution and information only conditions for any topic; however, the perception that the scientist was motivated to inform the public was lower in the controversial solution condition in comparison to the information only conditions for both the flu and marijuana topics.

For the perception that the scientist was *motivated by scientific evidence*, solution position only had an effect in the severe weather topic. Individuals exposed to the non-controversial solution were more likely to believe that the scientist was motivated by their evaluation of the scientific evidence in comparison to the information only topic.

Finally, for the motivations of *personal promotion* and *political views*, the controversial solution was higher, than the information only condition for the flu topic. There were no other observed differences for those two motivations across the other topics.

To examine RQ3, H1, and H2, we looked at the relationship between the six motivations and credibility (see Table 4 for all results). We predicted that the motivations that exhibited a benefit to the public (i.e., scientific evidence, informing the public, serving the public) would positively predict credibility, which was confirmed, thus H1 was supported. Perception of motivation to persuade the public was positively related to credibility for two out of four topics, regarding RQ3. Perception that the scientist was motivated by personal promotion was negatively related to credibility across all four topics. However, contrary to H2, perception of

Table 3. Effect of solution position on perceived motivations of the scientist.

	Scientific Evidence	Inform Public	Serve Public	Persuade the Public	Personal Promotion	Political Views
Non-Controversial vs. Information Only						
Flu	0.12	0.18	0.41**	0.58***	0.01	-0.12
Marijuana	0.03	0.04	0.13	0.36*	-0.20	0.04
Severe Weather	0.33*	-0.14	0.43***	1.19***	-0.01	-0.14
Climate Change	0.12	0.14	0.35*	0.60***	-0.19	0.29
Controversial vs. Information Only						
Flu	-0.13	-0.68***	-0.37**	0.50***	0.31+	0.54**
Marijuana	0.07	-0.43*	-0.15	0.31*	0.02	0.17
Severe Weather	0.20	-0.28	0.35**	1.25***	0.11	0.20
Climate Change	-0.03	-0.17	0.03	0.37**	0.04	0.33

Note: Entries are unstandardized regression coefficients and can be interpreted as the difference in means between the two conditions.

$^{+}$ $p < .10$,

* $p < .05$,

** $p < .01$,

*** $p < .001$

https://doi.org/10.1371/journal.pone.0187511.t003

motivation because of political views was not a significant predictor across any topic. Taken together, these results indicate that H2 was partially supported.

Results of the mediation analysis (RQ4) indicated that some motivations did mediate the effects of solution position on perceived credibility (see Table 5 for all coefficients). Most notably, the indirect effects through the perception that the scientist was *motivated to serve the public* were positive and significant for the non-controversial solution position (vs. the information-only condition) in 3 out of 4 topics (marijuana was the exception). This mediator was also significant for two out of four topics when looking at the indirect effects of the controversial vs. information only condition. For the topic of flu, the indirect effect was negative, such that the controversial flu solution resulted in decreased perceptions of serving the public, which in turn decreased credibility. For the topic of severe weather, the indirect effect was

Table 4. Perceived motivations relationships with credibility.

	Credibility			
	Flu	Marijuana	Severe weather	Climate change
Scientific evidence	0.26***	0.32***	0.35***	0.30***
Inform public	0.09***	0.18***	0.06*	0.16***
Serve public	0.41***	0.36***	0.26***	0.33***
Persuade public to take action	0.12***	0.03	0.06	0.11***
Personal promotion/gain	-0.08**	-0.12***	-0.08***	-0.09***
Political views	-0.02	-0.05	-0.03	0.00

Note: Entries are unstandardized regression coefficients from regressions predicting credibility from the motivations; one regression was conducted for each topic.

*p < .05,

**p < .01,

***p < .001

https://doi.org/10.1371/journal.pone.0187511.t004

Table 5. Indirect effects of solution position on credibility, through perceived scientist motivations.

	Indirect Effect on Credibility					
	**Via scientific evidence**	**Via inform the public**	**Via serve the public**	**Via persuade the public**	**Via personal promotion**	**Via political views**
Non-Controversial vs. Information Only						
Flu	0.03	0.02	0.17*	0.07*	0.00	0.00
Marijuana	0.01	0.01	0.04	0.01	0.02	0.00
Severe Weather	0.11*	-0.01	0.11*	0.07	0.00	0.00
Climate Change	0.04	0.02	0.11*	0.06*	0.02	0.00
Controversial vs. Information Only						
Flu	-0.03	-.06*	-0.15*	0.06*	-0.03	0.00
Marijuana	0.02	-.08*	-0.05	0.01	0.00	0.00
Severe Weather	0.07	-.02*	0.09*	0.08	0.00	0.00
Climate Change	0.00	-0.03	0.00	0.04*	0.00	0.00

Note: Entries are unstandardized indirect effects, generated by the PROCESS macro (Hayes, 2013). See Table 2 for Total Effects.

*$p < .05$

https://doi.org/10.1371/journal.pone.0187511.t005

positive, the controversial solution increased perceptions of serving the public, which in turn increased perceptions of credibility. Thus, overall, the perception that the scientist was motivated to serve the public was bolstered when he offered a non-controversial solution, which increased his credibility. There was no clear pattern of the effect of controversial solutions on the perception that the scientist was motivated to serve the public; however, this perceived motivation was consequential to perceptions of credibility.

The indirect effects through the perception that the scientist desired to *persuade the public* were positive and significant for both the non-controversial and controversial solution positions for 2 out of 4 topics (flu and climate change). For these two topics, offering any solution (controversial or not) increased the perception that the scientist wanted to persuade, which in turn, increased the perception that he was credible.

The indirect effects through the mediator of *informing the public* was significant only for the controversial solution position (but not for the non-controversial positions). For 3 out of the 4 topics, the controversial solution decreased the perception that the scientist desired to inform the public, which in turn decreased credibility. Thus, overall, offering a controversial solution decreased the perception that the scientist was motivated by a desire to inform the public, which subsequently lowered his credibility.

Finally, for the mediator of being *motivated by scientific evidence*, the non-controversial solution position had a positive indirect effect for one topic (severe weather), where offering a non-controversial solution increased the perception that the scientist was motivated by evidence, which increased credibility. There were no significant indirect effects on credibility through the mediators of *personal promotion* or *political views*.

Discussion

The purpose of this study was to examine the effect of advocacy on credibility of scientists, and to study the mediating role that perceived motivations of scientists play in explaining the relationship between scientist advocacy positions and credibility. Results from our study indicate that advocacy for non-controversial policies benefited credibility in some cases, and never harmed credibility compared to when a scientist simply provided information on the risks for

a scientific issue. Results for advocacy of controversial solutions were mixed; for the issue of the flu there was a negative overall effect on credibility, while for the issue of severe weather there was a positive effect, with no difference observed for the issues of climate change or marijuana use.

In sum, our findings contradict the main argument against science advocacy, which is that scientist advocacy is inevitably detrimental to scientists' credibility [4, 27]. Our results exemplify that some advocacy positions–those that receive broad support and are seen as "non-controversial"–can actually lead to increased public perception of scientists' motivations as benevolent, as well as higher levels of credibility among the public. Additionally, for one topic the topic of severe weather, even advocating for the controversial solution resulted in increased credibility (but was negative for the flu topic, and non-significant for two topics).

Our decision to test these effects across various topics in science communication was a way to assess the external validity of our results. What emerged is that the effects of solution position seemed more dependent on the topic studied and the specific solutions proposed, than on the level of controversy attributed to the message. For example, the controversial flu solution suggested everyone in the U.S. be mandated to get a vaccination whereas the controversial severe weather solution restricted coastal development. While both were perceived as more controversial compared to their non-controversial counterparts in our pre-test and both involve restrictions on individual freedom (i.e., freedom over one's body and freedom over one's property), personal vaccine mandates may feel more personally relevant for a greater number of people than restrictions on coastal development. This is because many people do not own property near the coast and may have no intentions to own coastal property, whereas a nationwide vaccine requirement would apply to everyone.

Further, non-controversial solutions resulted in increased credibility for climate change and severe weather compared to information-only conditions, but did not influence credibility for the flu and marijuana topics. This could be because participants viewed the climate change and severe weather non-controversial solutions (tax rebates for solar panels and severe weather alerts, respectively) as more effective than those for flu and marijuana (informational signs and warning labels, respectively). It may also be that earth science topics, such as weather and climate are simply seen as less personal than health science topics, such as flu and marijuana use, and therefore scientists may be given more latitude in prescribing solutions. The variation in our findings indicate that issue context–and the specific solution proposed, even within a broader category, such as "non-controversial"–may play an important role in how the public perceive scientists' motivations. Although we did not predict the relationship that scientist position advocacy has on credibility would vary by topic, it is useful to know that the public may respond differently according to characteristics of the topic studied.

Of course, the public may also respond differently to scientific communication depending on their personal characteristics. In response to suggestions by reviewers, we examined political ideology as a moderating variable. Few of the omnibus interactions were significant and the results did not explain the disparate findings between topics (see S1 Table). Although our analyses showed that political ideology did not significantly alter the results presented here, future research should examine how other demographic variables may influence how individuals perceive scientists who advocate. However, the inconsistent results across topics can influence the practical implications of the findings. Future research should carefully consider the intersection of issue context and individual characteristics to understand why perceptions of scientists who advocate may differ.

Our study shows that non-controversial solutions generally resulted in higher scientist credibility than providing information only, which could change how scientists communicate with the public about threatening issues. This lends support to the claim that scientist advocacy

is not inherently a bad thing and may not repel the public; rather non-controversial solutions may be the best avenue to utilize when communicating science issues to the public. This may also indicate that the public desires expert guidance about how to address risks, not simply to learn that risks exist, which could increase self or collective efficacy to address a problem. Further, some have suggested that researchers are expected to discuss the health risks associated with non-contentious issues, such as smoking and obesity, and to promote healthy behaviors in our society [28]. However, it is necessary to establish how people decide if a solution is controversial or not. Future research should systematically examine what factors contribute to a policy being perceived as non-controversial and potentially better operationalize the distinction between non-controversial and controversial solutions. For example, future studies could examine how the perceived effectiveness of the solution influences whether one deems it "controversial." Individuals may believe that signage is an acceptable and effective solution to preventing the flu, while for climate change prevention to be effective they may believe it warrants a more personally invasive course of action (i.e., carbon tax, driving restrictions). Therefore, increased perceived policy response efficacy may result in decreased perceived controversy of a solution.

Of course, in some cases, a scientist may believe that a controversial solution is the best option for addressing some risk. Our study suggests that advocating for such a position *may* have negative consequences to a scientist's credibility (although it is important to temper this claim as there was only a negative total effect on credibility for a controversial solution in 1 out of 4 topic domains included in this study; indeed, in one case there was a positive effect on credibility for a controversial solution; corroborating the finding in the Kotcher et al. study that some policy solutions may have slight negative effects on credibility, while others may not [12]). Future research should examine whether scientists can employ rhetorical strategies that might reduce the potential negative consequences of advocating for a controversial solution. Additionally, future research could provide important suggestions about boundary conditions under which advocating for non-controversial or controversial solutions are likely to harm scientific credibility.

Additionally, this study investigates the effect of perceived motivations on the credibility of the scientist. We found that when people perceived that the scientist shared information because of his desire to serve the public, inform the public, and as a result of his evaluation of the scientific evidence, they tended to rate that scientist as more credible for each topic studied. This corroborates previous research that found scientists who were perceived as benefiting society have higher public trust than scientists who appear to be self-interested [20]. Additionally, we found that, for two topics (climate change and flu), the perception that the scientist desired to persuade the public was associated with increased credibility (and for no topic was this motivation associated with decreased credibility), reinforcing skepticism about the inherent downsides of scientific advocacy.

Our results indicate that the motives the public ascribe the scientist are important to their credibility assessment of the scientist. To improve their credibility, scientists should focus on how to present themselves in a way that will highlight their positive motivations (scientific evidence, informing the public, and serving the public) for sharing information or advocating for a certain solution rather than negative motivations (personal promotion). It is possible that by including the reasoning for communicating with the public about scientific issues, individuals may believe the scientist is more credible, therefore accepting the information and changing their attitudes. Future research can help determine more motivations that influence scientist credibility, as well as further examine the motives we presented in this paper to see which are the most prominent influences on credibility (both positive and negative).

Our study has numerous limitations. First, although we sought to provide experimental control by standardizing much of the wording of the various experimental conditions, balancing experimental control with external validity concerns (that the advocacy positions be matched to the risks presented) resulted in advocacy positions that were non-equivalent in some aspects across the four topics tested. For example, the controversial solution for the flu issue was a personally intrusive suggestion that flu shots should be mandatory for every person. The controversial position for marijuana, on the other hand, suggested there be legislation that restricted medicinal marijuana use, which was unlikely to affect all participants, nor is it as medically invasive. We acknowledge that this difference could explain why the flu controversial position was the only condition to have a negative relationship with the scientist's credibility. In the future, researchers could attempt to find solutions that are more balanced across topics.

Additionally, it is important to note other limitations in the experimental design. First, the limitations of a non-probability, quota sample should be noted. Although the sample was matched to national benchmarks on gender, age, and education, the non-random selection can influence the generalizability of the results. It may be that those who sign up for survey panels are generally more amenable to the scientific endeavor, for example, than the rest of the US population. However, quota matching a non-probability sample to national benchmarks is superior to other non-probability methods. Second, participants saw only a blurb from an op-ed that contained the solution or information. There was no context surrounding the message and there was no counter-message, which limits external validity. When seeing scientific advocacy messages in a natural setting, individuals might be exposed to competing viewpoints regarding one issue. An opportunity for future research is to develop counter-messages to the advocacy position that the scientist is taking on a specific issue. Such research would get a more realistic sense of how the participants perceive the scientists' motivations in advocating or sharing information in the context of counter claims. Further, indicators for the perceived motivations were all single-item, which makes assessing the reliability of the measure more difficult. Future studies should develop multiple items that could measure the perceived motivations.

Third, as noted in our methods section, the credibility measure included in the questionnaire had eight radio buttons from which the participant could choose, but was labeled from 1–7 (the labels appeared in-between the radio buttons, see S1C). This programming error could have implications for the validity of this study. Although the visual representation of the question may have influenced the respondent's ability to accurately report his/her evaluation of the scientist, we argue that semantic differential scale relies on the proximity of the respondent's choice to each side of the adjective pairs rather than the exact values that the participant reports (e.g., 1 = strongly disagree, 7 = strongly agree). In other words, participants could still express the relative degree to which they thought Dr. Wilson was credible even with eight radio buttons instead of seven. Moreover, as this error was consistent across conditions, differences in credibility are still likely attributed to our experimental manipulations.

Finally, our study used mediation analysis to examine the influence that the perceived motives had on the relationship between position advocacy and credibility. Although participants were randomly assigned to the position advocacy condition, they were not randomly assigned to the mediating variables, which hinders our ability to predict a causal relationship regardless of the statistical significance of the model. Future research should consider the benefits of using a manipulation-of-mediator experimental design rather than the typical measurement-of-mediation experimental design [29].

As scientists consider advocating for certain issues, it is important to remember that the public's perceptions of the scientist's motivations is a meaningful predictor of credibility. This

corroborates Nelson and Vucetich's opinion that scientists have a duty to be transparent and just in their advocacy, which will likely improve credibility. While trust in science and scientists remains fairly high [30, 31], it has declined over the past decades, especially with the politicization of numerous science and public health issues [31]. It is crucial for scientists to gain trust and support of the public to help society. Our research suggests that scientists do have latitude to encourage specific issue solutions, but that they should be cognizant of how the public may perceive their motivations and emphasize the reasons they are supporting particular actions.

Supporting information

S1 Appendix. Questionnaire and experimental design.
(DOCX)

S1 Table. Analysis moderated by political ideology.
(DOCX)

Author Contributions

Conceptualization: Teresa A. Myers, John E. Kotcher, Emily K. Vraga, Edward W. Maibach.

Data curation: Teresa A. Myers.

Formal analysis: Teresa A. Myers.

Funding acquisition: Edward W. Maibach.

Methodology: Teresa A. Myers, John E. Kotcher, Emily K. Vraga.

Resources: Edward W. Maibach.

Writing – original draft: Lindsey Beall, John E. Kotcher.

Writing – review & editing: Lindsey Beall, Teresa A. Myers, John E. Kotcher, Emily K. Vraga, Edward W. Maibach.

References

1. Anderson L, Betsill M. Scientists' perspectives on navigating the science-policy frontier. Proceedings of the APSA Annual Meeting; 2010 Sep 2–5; Washington, DC.
2. Singh GG, Tam J, Sisk TD, Klain SC, Mach ME, Martone RG, et al. A more social science: barriers and incentives for scientists engaging in policy. Front Ecol Environ. 2014; 12: 161–66. https://doi.org/10.1890/130011
3. Lach D, List P, Steel B, Shindler B. Advocacy and credibility of ecological scientists in resource decisionmaking: a regional study. Biosci. 2003; 53: 170–78. https://doi.org/10.1641/0006-3568(2003)053[0170:aacoes]2.0.co;2
4. Lackey RT. Science, scientists, and policy advocacy. Conserv Biol. 2007; 21: 12–17. https://doi.org/10.1111/j.1523-1739.2006.00639.x PMID: 17298504
5. Pielke RA. The honest broker: making sense of science in policy and politics. Chicago: Cambridge University Press; 2007
6. Donner SD. Finding your place on the science–advocacy continuum: an editorial essay. Clim Change. 2014; 124: 1–8. https://doi.org/10.1007/s10584-014-1108-1
7. Nisbet EC, Coope KE, Garrett RK. The partisan brain how dissonant science messages lead conservatives and liberals to (dis)trust science. Ann Am Acad Pol Soc Sci. 2015; 658: 36–66. https://doi.org/10.1177/0002716214555474
8. Nelson M, Vucetich JA. On advocacy by environmental scientists: What, whether, why, and how, Conserv Biol. 2009; 23: 1090–1101. https://doi.org/10.1111/j.1523-1739.2009.01250.x PMID: 19459889

9. Renn O, Levine D. Credibility and trust in risk communication. In: Kasperson RE, Stallen PJM, editors. Communicating risks to the public. Netherlands: Springer; 1991. pp. 175–217
10. Kasperson RE, Golding D, Tuler S. Social distrust as a factor in siting hazardous facilities and communicating risks. J Soc Issues. 1992; 48: 161–187.
11. Peters RG, Covello VT, McCallum DB. The determinants of trust and credibility in environmental risk communication. Risk Analysis. 1997; 17: 43–54. PMID: 9131825
12. Kotcher JE, Myers TM, Vraga EK, Stenhouse N, Maibach E. Does engagement in advocacy hurt the credibility of scientists? Results from a randomized national survey experiment. Environ Commun. 2017, forthcoming.
13. Reeder GD. Attribution as a gateway to social cognition. In: Calstron DE, editor. Oxford handbook of social cognition. Oxford: Oxford University Press; 2013. pp. 95–117.
14. Malle BF. Attribution theories: How people make sense of behavior. In: Chadee D, editor. Theories in social psychology. Malden, MA: Wiley-Blackwell. 2011. pp. 72–95.
15. Malle BF, Holbrook J. Is there a hierarchy of social inferences? The likelihood and speed of inferring intentionality, mind, and personality. J Pers Soc Psychol. 2012; 102: 661. https://doi.org/10.1037/a0026790 PMID: 22309029
16. Reeder GD. Mindreading: Judgments about intentionality and motives in dispositional inference. Psychol Inquiry. 2009; 20: 1–18. https://doi.org/10.1080/10478400802615744
17. Earle TC, Siegrist M, Gutscher H. Trust, risk perception, and the TCC model of cooperation. In: Trust in cooperative risk management: Uncertainty and scepticism in the public mind. London: Earthscan. 2007. pp. 1–49.
18. Fiske ST, Dupree C. Gaining trust as well as respect in communicating to motivated audiences about science topics. Proceedings of the National Academy of Sciences; 2014; 111 Supp l4:13593–13597. https://doi.org/10.1073/pnas.1317505111 2014 PMID: 25225372
19. Wood W, Eagly AH. Stages in the analysis of persuasive messages: The role of causal attributions and message comprehension. J Pers Soc Psychol. 1981; 40: 246–259. https://doi.org/10.1037/0022-3514.40.2.246
20. Critchley CR. Public opinion and trust in scientists: The role of the research context, and the perceived motivation of stem cell researchers. Public Underst Sci. 2008; 17: 309–327. https://doi.org/10.1177/0963662506070162 PMID: 19069082
21. Combs DJY, Keller PS. Politicians and trustworthiness: Acting contrary to self-interest enhances trustworthiness. Basic Appl Soc Psych. 2010; 32: 328–339. https://doi.org/10.1080/01973533.2010.519246
22. Eagly AH, Wood W, Chaiken S. Causal inferences about communicators and their effect on opinion change. J Pers Soc Psychol. 1978; 36: 424–435. https://doi.org/10.1037/0022-3514.36.4.424
23. Rabinovich A, Morton TA, Birney ME. Communicating climate science: The role of perceived communicator's motives. J Environ Psychol. 2012; 32: 11–18. https://doi.org/10.1016/j.jenvp.2011.09.002
24. Ruggiero LF. Scientific independence and credibility in sociopolitical processes. J Wildl Manag. 2010; 74: 1179–1182.
25. McCroskey JC, Teven JJ. Goodwill: A reexamination of the construct and its measurement. Commun Monogr. 1999; 66: 90–103. https://doi.org/10.1080/03637759909376464
26. Hayes AF. An introduction to mediation, moderation, and conditional process analysis: A regression-based approach. New York: Guilford Press; 2013.
27. Kaiser J. Ecologists on a mission to save the world. Science. 2000; 287: 1188–1192. https://doi.org/10.1126/science.287.5456.1188 PMID: 10712146
28. Corner A, Groves C. Breaking the climate change communication gridlock. Nat Clim Change. 2014; 4: 743–745. https://doi.org/10.1038/nclimate2348
29. Pirlott AG, MacKinnon DP. Design approaches to experimental mediation. J Exp Soc Psychol. 2016; 66: 29–38. https://doi.org/10.1016/j.jesp.2015.09.012 PMID: 27570259
30. Myers TA, Kotcher J, Stenhouse N, Anderson AA, Maibach E, Beall L, et al. Predictors of trust in the general science and climate science research of US federal agencies. Public Underst Sci. 2016: 096366251663604. https://doi.org/10.1177/0963662516636040 PMID: 26960910
31. Pew Research Center. Public and scientists' views on science and society. 2015; Washington, DC: Pew Research Center for the People & the Press
